# Compensatory Increase of Transglutaminase 2 Is Responsible for Resistance to mTOR Inhibitor Treatment

**DOI:** 10.1371/journal.pone.0149388

**Published:** 2016-02-12

**Authors:** Jingwen Cao, Wenlong Huang

**Affiliations:** 1 China Pharmaceutical University, Nanjing, Jiangsu Province, People's Republic of China; 2 Chinese Academy of Medical Sciences, Beijing, People's Republic of China; Yong Loo Lin School of Medicine, National University of Singapore, SINGAPORE

## Abstract

The mechanistic target of rapamycin complex 1 (mTORC1) plays a crucial role in controlling cell growth and homeostasis. Deregulation of mTOR signaling is frequently observed in some cancers, making it an attractive drug target for cancer therapy. Although mTORC1 inhibitor rapalog-based therapy has shown positive results in various pre-clinical animal cancer studies, tumors rebound upon treatment discontinuation. Moreover, several recent clinical trials showed that the mTORC1 inhibitors rapamycin and rapalog only reduce the capacity for cell proliferation without promoting cell death, consistent with the concept that rapamycin is cytostatic and reduces disease progression but is not cytotoxic. It is imperative that rapamycin-regulated events and additional targets for more effective drug combinations be identified. Here, we report that rapamycin treatment promotes a compensatory increase in transglutaminase 2 (TGM2) levels in mTORC1-driven tumors. TGM2 inhibition potently sensitizes mTORC1-hyperactive cancer cells to rapamycin treatment, and a rapamycin-induced autophagy blockade inhibits the compensatory TGM2 upregulation. More importantly, tumor regression was observed in MCF-7-xenograft tumor-bearing mice treated with both mTORC1 and TGM2 inhibitors compared with those treated with either a single inhibitor or the vehicle control. These results demonstrate a critical role for the compensatory increase in transglutaminase 2 levels in promoting mTORC1 inhibitor resistance and suggest that rational combination therapy may potentially suppress cancer therapy resistance.

## Introduction

The mammalian target of rapamycin complex 1 (mTORC1) is a master regulator of the cellular response to multiple signals including growth factors, nutrients, energy, and oxygen, and ultimately controls a variety of biological process including mRNA biogenesis; protein, lipid and nucleotide synthesis; energy metabolism; and autophagy [1–3]. Abnormal mTORC1 signaling activation is frequently observed in variety of tumors due to gain-of-function oncogene mutations (e.g., PI3K, AKT, and Ras), and/or tumor suppressor loss-of-function mutations (e.g. PTEN, LKB1, and TSC1/2), which are crucial upstream regulators of mTORC1 [4, 5]. Consequently, mTORC1 inhibitors such as rapamycin are considered to be beneficial in cancer therapy; however, recent clinical trials using mTORC1 inhibitors demonstrated that although these drugs promoted tumor shrinkage, the tumors rebounded upon treatment suspension [6, 7]. These observations highlight an immediate need for the identification of additional targets for more effective drug combinations. Herein, we have studied a subset of mTORC1-driven tumor cells using loss-of-function mutations in the Tuberous Sclerosis Complex (TSC) composed of tumor suppressor genes including *TSC1* and *TSC2*. The proteins encoded by the *TSC1* and *TSC2* genes, hamartin and tuberin, respectively, interact with TBC1 domain family member 7 (TBC1D7) to form an active complex that regulates the mTORC1 activation state [8–10]. Mutation and loss of either the *TSC1* or *TSC2* gene leads to mTORC1 hyperactivation. In this study, we demonstrated that according to bioinformatics analysis, rapamycin treatment promotes transglutaminase 2 (TGM2) expression in both *Tsc2*^-/-^ and *Tsc1*^-/-^ MEFs.

Transglutaminase 2 (TGM2) belongs to a family of transglutaminases, which are multifunctional enzymes that are ubiquitously expressed in mammalian tissues. TGM2 catalyzes Ca2^+^-dependent post-translational protein modifications by forming irreversible e-(c-glutamyl)-lysine cross-links between polypeptide chains. TGM2 is also known to possess multiple enzymatic activities including hydrolase, protein disulfide isomerase and protein kinase activities [11–13]. TGM2 is typically considered to be predominantly localized to cellular membrane compartments, the cytosol and nucleus; however, several studies have demonstrated the expression of a secreted form in some cells. TGM2 is also associated with several biological functions including apoptosis, signal transduction, cell migration, cell adhesion and extracellular matrix (ECM) [11, 12].

Here, we report that inhibition of transglutaminase 2 (TGM2), a compensatory response to rapamycin treatment, potently sensitizes mTORC1-hyperactive cancer cells to rapamycin treatment. Importantly, the combination of rapamycin and a TGM2 blockade promoted tumor regression in an MCF-7-xenograft tumor model. Consequently, our study reveals an attractive approach that could potentially be developed for clinical use against mTORC1-driven cancers and has the potential to improve the outcomes of cytostatic rapamycin-based therapies.

## Materials and Methods

### Gene expression analysis

The complete microarray data set is available at http://www.ncbi.nlm.nih.gov/geo (accession number GSE21755). Detailed protocols describing this gene expression array were described by Duvel et al. [14]. Expression levels in Tsc1-/- and Tsc2-/- cells treated with rapamycin for 2, 6, 12, and 24 h were compared with the corresponding vehicle-treated 0 h controls. Expression levels in vehicle-treated wild-type cells were compared with those of both the 0 and 24 h rapamycin time points for Tsc1-/- and Tsc2-/- cells. The expression levels of gene probes meeting four independent criteria at a p-value < 0.01 were calculated: (1) different in rapamycin-treated Tsc1^-/-^ cells versus the control; (2) different in rapamycin-treated Tsc2^-/-^ cells versus the control; (1) not reverted towards wild-type levels by rapamycin treatment of Tsc1^-/-^ cells; and (4) not reverted towards wild-type levels by rapamycin treatment of Tsc2^-/-^ cells.

### Quantitative RT-PCR

Total RNA was extracted using the RNeasy mini kit (Qiagen) and 1 μg was used to synthesize cDNA with a high-capacity cDNA reverse transcription kit (Life Technologies) according to the manufacturer's protocol. Gene expression was quantified using SYBR Green real-time PCR Master Mix kit (Life Technologies) in an Applied Biosystems Real-Time PCR System and normalized to Tubulin. The primers were human TGM2, TGTGGCACCAAGTACCTGCTCA (forward) and GCACCTTGATGAGGTTGGACTC (reverse); and mouse TGM2, GAAGGAACACGGCTGTCAGCAA (forward) and GATGAGCAGGTTGCTGTTCTGG (reverse).

### Cell culture and reagents

Cells were cultured in a humidified incubator at 37°C with 5% CO_2_. MEF cells were cultured in Dulbecco’s modified Eagle media (DMEM) containing 10% fetal bovine serum (FBS). MCF-7 and 786-O cells were cultured in RPMI-1640 media containing 10% FBS. Cells were treated with either 20 nM rapamycin, 500 μM KCC009 or a combination for 24 h; vehicle alone was used as a control. Rapamycin was purchased from Sigma-Aldrich. KCC009 ((S)-[3-(4-hydroxyphenyl)-2-N-(phenylmethyloxycarbonyl) aminopropanoic acid N0 -(30 -bromo-40,50—dihydro-50 -isoxalyl)methylamide) was prepared as previously described [15]. 1H NMR (CDCl3, 200 MHz): d = 7.34 to 7.26 (m, 8 H), 7.17 (d, 2 H, J = 7.6 Hz), 6.19 to 6.09 (m, 1 H), 5.21 to 5.15 (m, 1 H), 5.09 (s, 2 H), 4.74 to 4.60 (m, 1 H), 4.41 to 4.36 (m, 1 H), 3.49 to 3.45 (m, 2 H), 3.26 to 3.12 (m, 1 H), 3.07 (d, 2 H, J = 6.8 Hz), 2.97 to 2.76 (m, 1 H): MS (ESI) m/z 460.1 [M+H]+, 482.2 [M+Na]+. The compound was purified by SiO2 chromatography as a white solid (1 g, 55%).

### Cell viability assay

Cells were seeded in 96-well plates for 24 h at a density of 5 x10^3^/ml and then treated with either inhibitors or a vehicle control for 24 h. Cell viability was determined by an MTS assay (Promega) according to the manufacturer’s instruction.

### Cell death assay (flow cytometry)

Cells were seeded overnight in 6-well plates and then treated with a vehicle control, rapamycin, KCC009 or a combination of rapamycin and KCC009 for 24 h. Cells were harvested and stained with Annexin V:FITC (BD) according to the manufacturer’s instructions and analyzed by flow cytometry.

### RNA Interference

293T cells were transfected with TGM2-targeting or non-targeting shRNA vectors using Lipofectamine 3000 (Life Technologies). The cells were infected with lentivirus containing TGM2-targeting or non-targeting shRNAs. Cells were harvested 48 h after transfection, selected using puromycin and stable clones were harvested for future experiments. siRNAs were transferred using RNAiMAX (Life technologies) according to the manufacturer’s instructions. Sequences were as follows: Tgm2 shRNA-1, 5′-AGGAGCTGGTCTTAGAGAGGTGTGATCTG-3′; Tgm2 shRNA-1, 5′-GACAAGAGCGAGATGATCTGGAACTTCCA-3′; Atg5 siRNA, 5′- ACCGGAAACUCAUGGAAUATT -3′; mTOR siRNA, 5′-GAACTCGCTG ATCCAGATG-3′; and control siRNA, 5′-TTCTCCGAAC GTGTCACGT-3′.

### TGM2 overexpression

Human TGM2 gene was cloned from human cDNA library and primers were designed according to the cDNA sequence (NM_004613.2). Mouse TGM2 gene was cloned from mouse cDNA library and primers were designed according to the cDNA sequence (NM_009373.3). All the cDNA were subcloned to pcDNA 3.1. The plasmids were transfected with lipofectamine 3000 (Thermo Fisher Scientific) according to manufacturer’s instruction.

### Animal studies

The protocol for our study was approved by the Animal Studies Committee at the China Pharmaceutical University. Animal experiments were performed in the animal facility at the China Pharmaceutical University according to governmental and institutional guidelines. Female CB17-SCID mice (8–10 weeks of age) were purchased from Vital River. MCF-7 cells were transduced with a luciferase tag for bioluminescent imaging. To establish the xenograft tumors, mice were bilaterally inoculated in their posterior back regions with 1 x 10^6^ cells. Four weeks post inoculation, mice bearing subcutaneous tumors were randomized into four groups: vehicle control (n = 5; 10% DMSO i.p.), rapamycin (n = 5; 1 mg/kg/day i.p.), KCC009 (n = 5; 50 mg/kg/day in 10% DMSO i.p.) and rapamycin plus KCC009 (n = 5; 1 mg/kg/day and 50 mg/kg/day, respectively, i.p.). Drug treatments were initiated four weeks post-inoculation, and tumor growth was monitored weekly using non-invasive imaging with an IVIS platform (Perkin Elmer). All efforts were made to reduce the suffering of the animals and minimize the number of animals used in the study. Animal health was monitored five days/week during the entire tumor experiments. The endpoint of the xenograft tumor study was the onset of the clinical signs of pain/distress including 1) animals are in constant pain (hunched posture, sluggish movement); 2) bilateral tumors (two subcutaneous tumors/mouse) have caused inactivity, became ulcerated and/or larger than 15% of the animal’s body weight (tumor volume, 1,000 mm^3^); 3) animals have lost more than 20% of their body weight. All mice were euthanized by carbon dioxide (CO2) inhalation via compressed gas in response to the onset of the above distress. The mice were caged in a pathogen-free facility in groups of five or fewer mice and were fed laboratory autoclavable rodent diet and water ad libitum. 12 light/12 dark cycle is used in mouse room. Temperatures is between 18°C and 20°C, and humidity is between 40% and 50%.

### Immunohistochemistry

Histology sections were prepared from xenograft tumors harvested from mice treated with a vehicle control, rapamycin, KCC009, and rapamycin and KCC009 following 10% formalin fixation and cutting into five 4 mm sections in cassettes. Immunohistochemistry (IHC) was performed on paraffin-embedded 4 μm sections using antibodies against PCNA (Cell Signaling Technology 2586S). Slides were processed using the Life Technologies SuperPictures 3^rd^ Gen IHC kit according to the manufacturer’s instructions. Hematoxylin was purchased from Sigma-Aldrich and used as a counterstain.

### Western blotting

Following treatment, cells were lysed in ice-cold lysis buffer (50 mM Tris-HCl (pH 7.5), 150 mM NaCl, 1.0% Triton X-100, 20 mM EDTA, 1 mM Na_3_VO_4_, 1 mM NaF and protease inhibitors). Lysates were cleared of insoluble material by centrifugation at 10,000g for 10 min at 4°C. The cell extract protein concentrations were measured using the Bradford assay (BioRad, 500–0006) according to the manufacturer’s protocol and a BioTek plate reader. Ten micrograms of protein were separated by polyacrylamide gel electrophoresis. The following antibodies were used: TGM2 (Abcam, ab421), p-S6 (Ser 235/236, Cell Signaling #2211), p-p70 S6 Kinase (Cell Signaling #9205), β-actin (Sigma, A5441), LC3 (Sigma, L8918), and cleaved-caspase 3 (Cell signaling #9661).

### Statistical analysis

All in vitro data are shown as means ± S.D and in vivo data are shown as means± S.E.M. Measurements at single time points were analyzed by ANOVA and then using a two-tailed t-test (Student’s t test). Time courses were analyzed by repeated measurements (mixed model) ANOVA and Bonferroni post-t-tests. Survival data were analyzed using Kaplan-Meier survival analysis. All statistical tests were performed using GraphPad Prism 5.0 (GraphPad Software, San Diego, CA, USA) and p< 0.05 indicated statistical significance.

## Results

### Transglutaminase 2 is regulated by rapamycin in Tsc2^−/−^ MEFs

**Fig 1:**
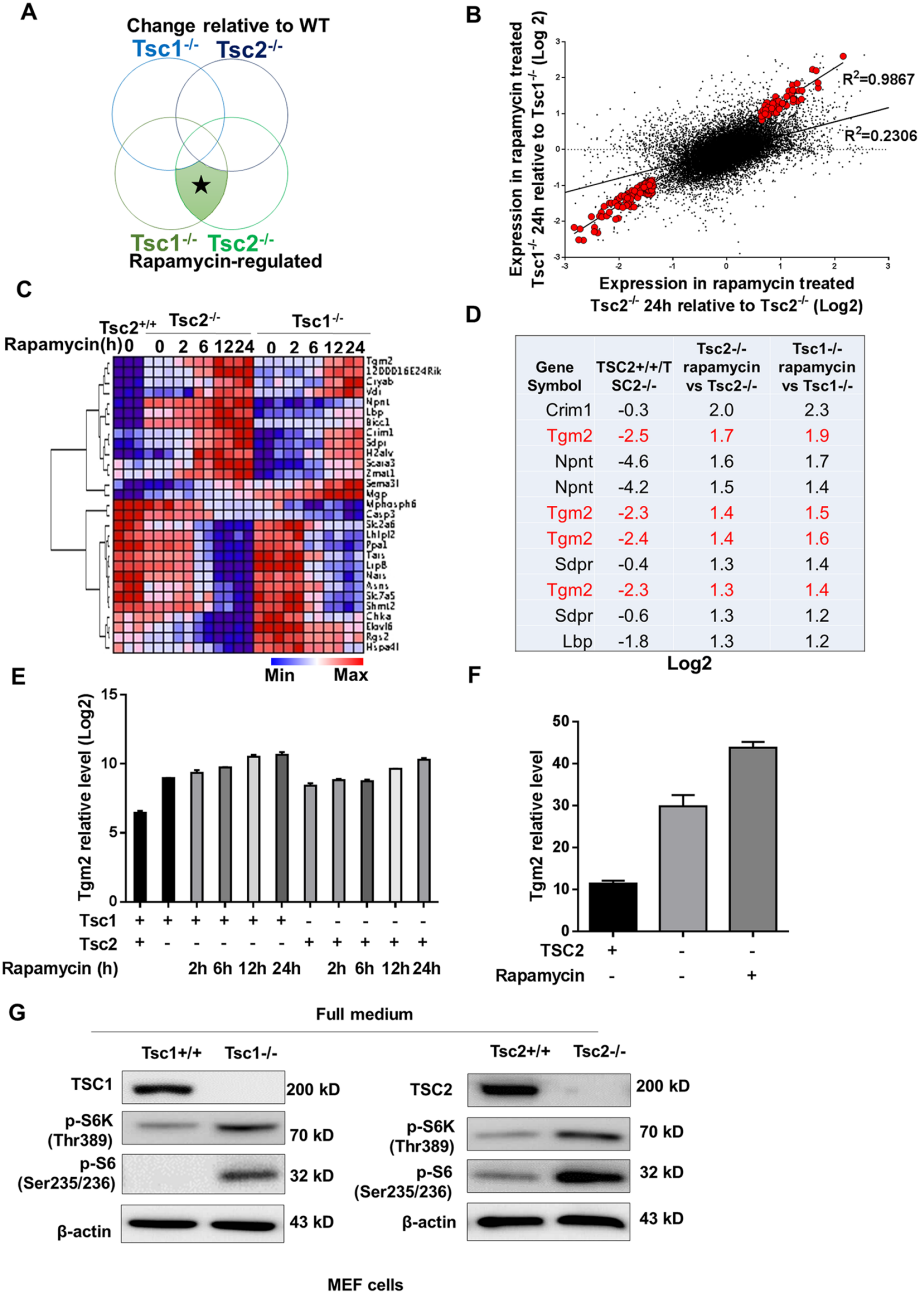
Rapamycin regulates Transglutaminase 2 in Tsc2−/− MEFs. (A) Hypothetical Venn diagram of the two major changes in gene expression observed in this study. Rapamycin-regulated transcripts were classified as those that met all four criteria at a statistical cut-off *p < 0.01. (B) Scatter plot of the expression levels (log_2_) of the 39,000 probed genes comparing rapamycin- and vehicle-treated Tsc2^-/-^ cells (x-axis), and rapamycin- and vehicle-treated Tsc1^-/-^ cells (24 h) (y-axis). The larger red dots indicate the expression pattern of the 29 gene probes meeting the criteria described in (A). The gray dots indicate the expression pattern of the entire dataset. (E) Heat map of the 29 rapamycin-regulated probes identified in this study showing their expression levels and regulation in response to rapamycin over time in WT, Tsc2^-/-^ and Tsc1^-/-^ cells. The expression levels are representative of the log_2_ value per sample. (D) Top 10 probes of rapamycin-regulated genes as well as those that were not reverted towards wild type levels by rapamycin treatment in TSC1 and TSC2-deficient cells. (E) Tgm2 transcript levels were compared among WT, Tsc2^-/-^ and Tsc1^-/-^ cells using the log_2_ values from the GSE21755 GEO dataset. (F) Tgm2 transcript levels were compared between TSC2-deficient (TSC2-) and TSC2-addback (TSC2+) cells, and rapamycin- and vehicle-treated TSC2-deficient cells (Rapa+ TSC2-) from the GSE16944 GEO dataset. (G) Immunoblots of TSC1, TSC2, phospho-S6 and phospho-p70 S6K in Tsc1^+/+^, Tsc1^-/-^, Tsc2^+/+^ and Tsc2^-/-^ MEF cells. All data are shown as means ± S.D. ** P < 0.01, *P < 0.05, Student’s t-test.

To identify rapamycin-enhanced transcriptional changes, a public GEO dataset focusing on rapamycin-treated *TSC*-deficient cells was re-analyzed. We compared rapamycin-treated *Tsc1*^-/-^ and *Tsc2*^-/-^ MEFs to non-treated cells, as loss of the tumor suppressor gene fully activates mTORC1 signaling in both cell types. To be identified as a rapamycin-enhanced transcript in this study, gene probes needed to meet four independent criteria at a p < 0.01: (1) different in rapamycin vs. control Tsc1^-/-^ cells; (2) different in rapamycin-treated vs. control Tsc2^-/-^ cells; (3) not reverted towards wild type levels by rapamycin in Tsc1^-/-^; and (4) not reverted toward wild-type levels by rapamycin in Tsc2^-/-^ cells (Fig 1A). From 39,000 genes, we identified 169 meeting conditions (1) and (2) that the transcript changed in the same trend in both rapamycin-treated Tsc1^-/-^ and Tsc2^-/-^ MEFs compared with the control. The scatter plot of these genes (red dots) relative to all others (gray dots) is shown in Fig 1B. These 169 genes included both those genes that were reduced by rapamycin treatment in TSC1 and TSC2-deficient cells (107 genes) and those that were stimulated by rapamycin (72 genes) (Table in S1 Table). To meet conditions (3) and (4), we examined these 169 genes over time in response to rapamycin treatment of wild type MEF cells (Fig 1C). Genes that were induced by rapamycin and were not reverted towards wild type levels by rapamycin in TSC1 and TSC2-deficient cells were chosen for further study. The top 10 probes meeting these conditions are shown in Fig 1D. One of the top hits, Tgm2, is highlighted because it appeared five times in the top twenty probes. By examining Tgm2 transcript levels in TSC1- and TSC2-deficient cells, we found that Tgm2 levels increased in a time-dependent manner in response to rapamycin treatment; however, they decreased in wide type MEFs (Fig 1E). Further study using patient-derived TSC2-deficient cells for expression analysis revealed a similar trend, in that Tgm2 transcript levels were increased in TSC2-deficient cells and even promoted by rapamycin treatment (Fig 1F). TSC1 and TSC2 expression levels were examined by western blot to confirm the identity of the Tsc1-/- and Tsc2-/- MEFs (Fig 1G). In addition to the absence of the respective protein, phosphorylation of p70 S6K and S6 were checked to indicative the constitutive activation of mTORC1 in both of cell lines (Fig 1G).

### Rapamycin treatment promotes transglutaminase 2 expression

**Fig 2:**
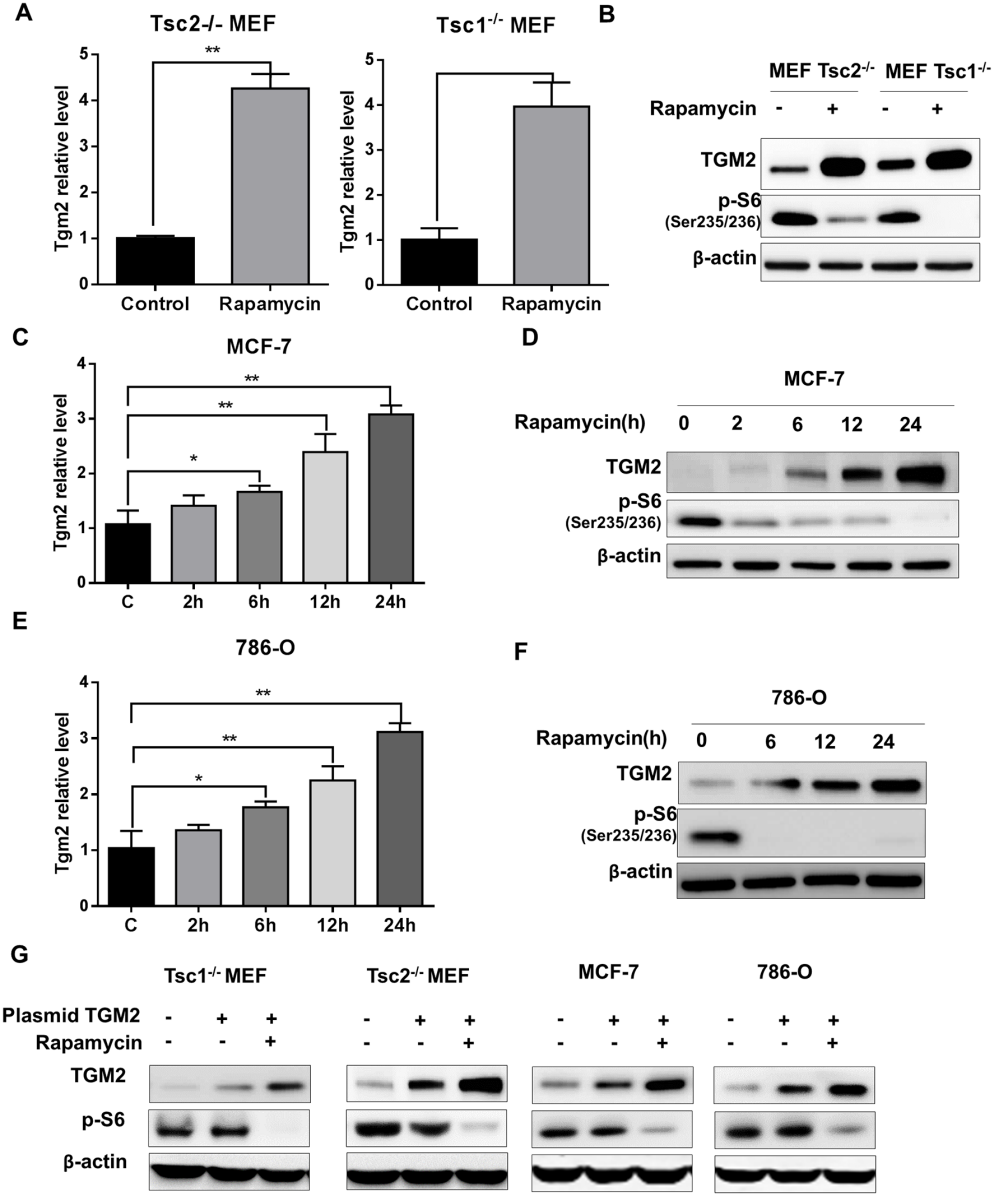
Rapamycin treatment promotes expression of transglutaminase 2. (A) Comparison of Tgm2 transcript levels in Tsc2^-/-^ and Tsc1^-/-^ MEF cells treated with either vehicle or rapamycin (n = 3). (B) Immunoblots of TGM2 and phospho-S6 in Tsc2^-/-^ and Tsc1^-/-^ MEF cells treated with either vehicle or rapamycin. (C) Tgm2 transcript levels in rapamycin-treated MCF-7 cells at various time points (n = 3). (D) Immunoblots of TGM2 and phospho-S6 in vehicle- and rapamycin-treated MCF-7 cells at various time points. (E) Tgm2 transcript levels in rapamycin-treated 786-O cells at various time points (n = 3). (F) Immunoblots of TGM2 and phospho-S6 in vehicle- and rapamycin-treated 786-O cells at various points. (G) Immunoblots of TGM2 and phosphor-S6 in in vehicle- and rapamycin-treated Tsc1^-/-^ and Tsc2^-/-^ MEFs, MCF-7 and 786-O cells transfected with vehicle or TGM2 plasmids. All data are shown as means ± S.D. ** P < 0.01, *P < 0.05, Student’s t-test.

We performed real-time PCR analysis to validate the observed increase in Tgm2 transcript levels in mTORC1-hyperactive cells. In the presence of rapamycin, relative Tgm2 levels were approximately four-fold increased in both TSC1- and TSC2-deficient cells (Fig 2A). Importantly, rapamycin promoted TGM2 protein expression in both TSC1- and TSC2-deficient MEF cells (Fig 2B). Phosphorylation of ribosomal protein S6 was shown to indicate mTORC1 suppression by rapamycin. To determine whether rapamycin treatment promotes TGM2 expression in other cancer cell lines, we measured Tgm2 transcript levels in MCF-7 (PTEN-mutant breast cancer) and 786-O (PI3K-mutant renal cancer) cells. Rapamycin treatment promoted Tgm2 transcript levels in both MCF-7 and 786-O cells in a time-dependent manner (Fig 2C and 2E). More importantly, TGM2 protein levels were also substantially increased in response to mTORC1 inhibition (Fig 2D and 2F). To further demonstrate increase of resistance to rapamycin may correlate to expression level of TGM2 protein, we overexpressed TGM2 protein by transfection in Tsc1^-/-^ and Tsc2^-/-^ MEFs, MCF-7 and 786-O cells. We found that expression level of TGM2 could be elevated by transfection of TGM2 plasmid, and rapamycin treatment even enhances the TGM2 expression level (Fig 2G). Our data suggest that TGM2 upregulation is a common mechanism for rapamycin-triggered events in mTORC1-hyperactive cancer cells. We therefore hypothesized that combined targeting of both mTORC1 and TGM2 would promote cancer cell death.

### Combined inhibition of TGM2 and mTORC1 reduced proliferation and viability

**Fig 3:**
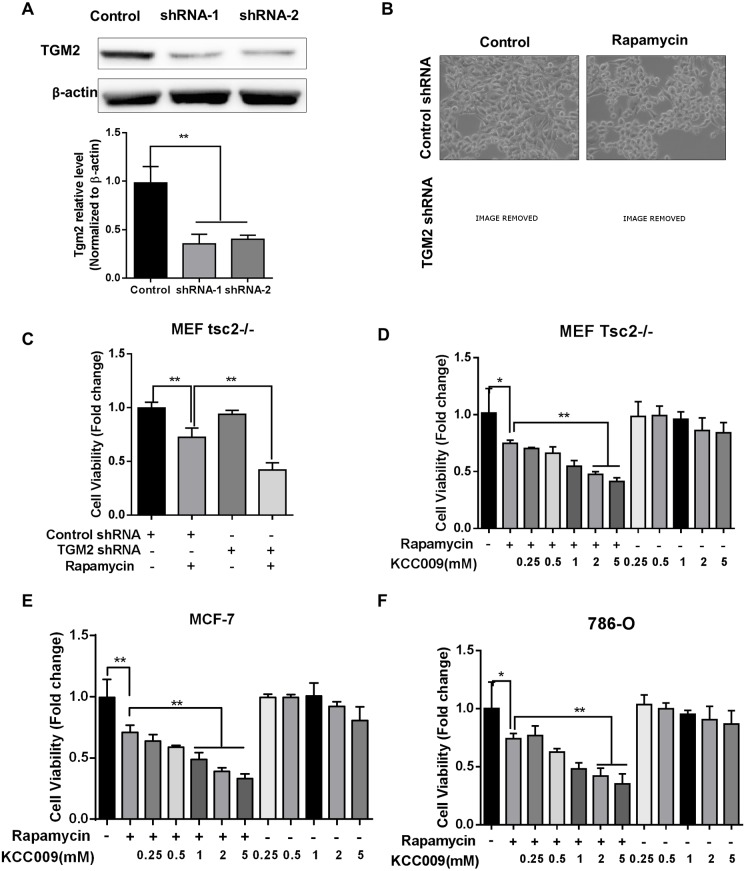
Combined TGM2 and mTORC1 inhibition leads to morphological changes and reduced viability. (A) Tsc2^-/-^ MEF cells were treated with siRNAs against TGM2 and lysates were used for immunoblotting. Densitometry is shown in the bar chart (n = 3). (B) Tsc2^-/-^ MEF cells were treated with either TGM2 siRNA or 20 nM rapamycin for 24 hours and cell morphologies were recorded using phase-contract microscopy. (C) Tsc2^-/-^ MEF cell viability following treatment with TGM2 siRNA and 20 nM rapamycin (n = 8). (D) Tsc2^-/-^ MEF cell viability following treatment with 20 nM rapamycin and the indicated doses of TGM2 inhibitor KCC009. (E) Viability of MCF-7 cells treated with 20 nM rapamycin and the indicated doses of TGM2 inhibitor KCC009 (n = 8). (F) Viability of 786-O cells treated with 20 nM rapamycin and the indicated doses of TGM2 inhibitor KCC009 (n = 8). All data are shown as means ± S.D. ** P < 0.01, *P < 0.05, Student’s t-test.

To assess the rationale of combined treatment, we first knocked-down TGM2 expression using shRNAs in tsc2^-/-^ MEF cells. Using western blotting, we verified that TGM2 was sufficiently suppressed by the shRNA (Fig 3A). Control shRNA- and TGM2 shRNA-treated tsc2^-/-^ MEF cells were then treated with rapamycin for 48 hours. Morphological observations revealed an obvious reduction in cell proliferation in response to combined mTORC1 and TGM2 inhibition (Fig 3B). Cell viability was assayed to verify the inhibition of both mTORC1 and TGM2 in tsc2^-/-^ MEF cells. TGM2 knockdown enhanced rapamycin-reduced cell viability compared with rapamycin only (Fig 3C). Additionally, we assayed the efficacy of combining rapamycin and the TGM2-specific inhibitor KCC009 in treating mTORC1-hyperactive cells. We found that 0.5 and 1 mM KCC009 in combination with rapamycin sufficiently reduced tsc2^-/-^ MEF cell viability (Fig 3D); similar results were observed for MCF-7 and 786-O cells (Fig 3E and 3F).

Our preliminary data demonstrate that combined rapamycin treatment and TGM2 blockade could be beneficial to mTORC1-hyperactive cancer cell treatment.

### TGM2 and mTORC1 inhibition potently induce Tsc2^−/−^ cell apoptosis

**Fig 4:**
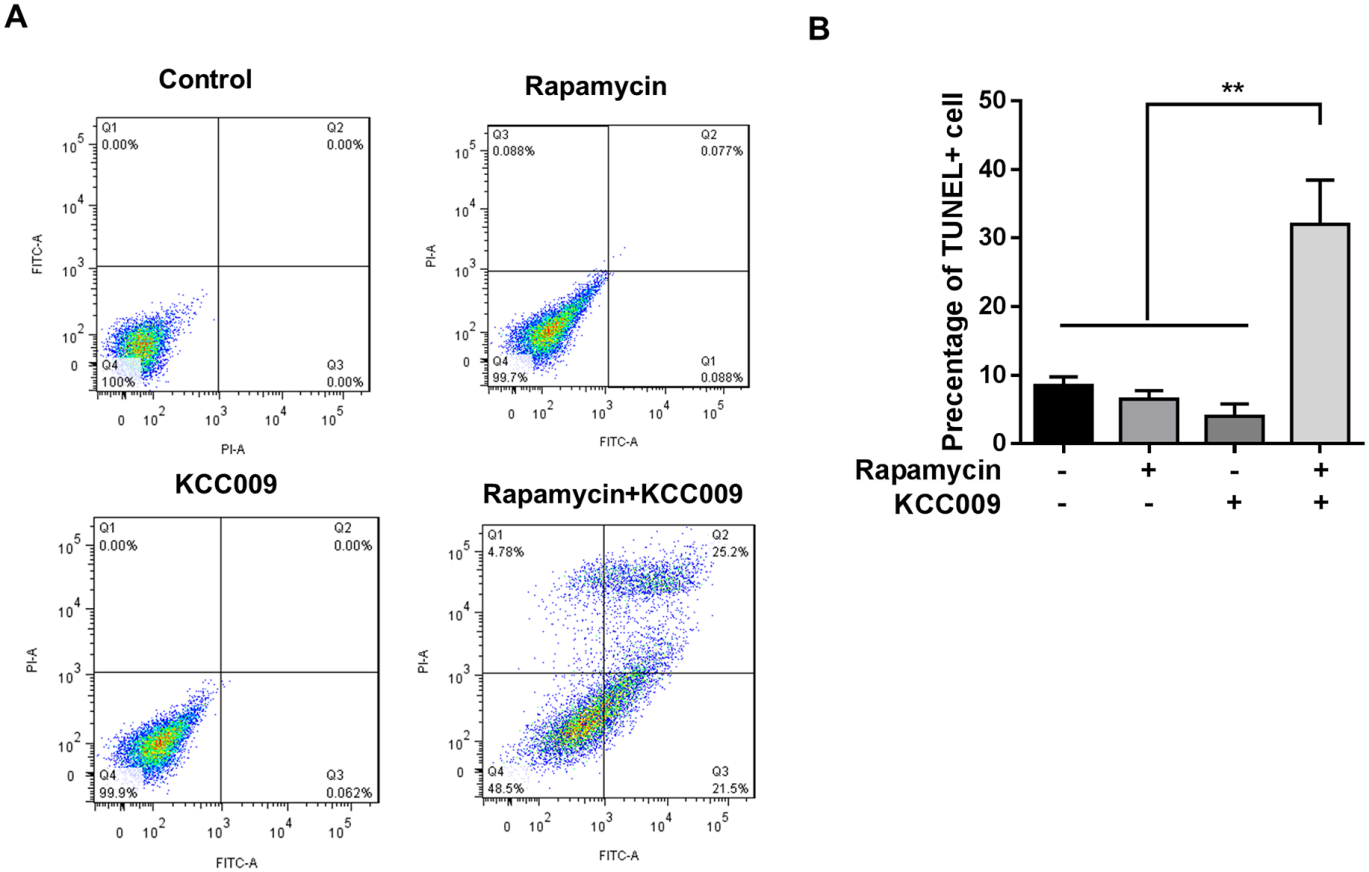
TGM2 and mTORC1 inhibition causes potent Tsc2−/− cell apoptosis. Tsc2^-/-^ MEF cells were treated with 20 nM rapamycin and 0.5 mM TGM2 inhibitor KCC009 for 24 hours. (A) Cell death was analyzed using PI and ANNEXIN V staining and flow cytometry. (B) Cell death was analyzed by TUNEL staining. Percentage of TUNEL+ cells is indicated in the bar chart (n = 4). All data are shown as means ± S.D. ** P < 0.01, *P < 0.05, Student’s t-test.

To determine whether the dual inhibition of mTORC1 and TGM2 could induce cancer cell apoptosis, we analyzed PI and ANNEXIN V staining of tsc2^-/-^ MEF cells by flow cytometry. This analysis demonstrated that treatment with either rapamycin or KCC009 alone failed to induce apoptosis, while treatment with both rapamycin and KCC009 obviously promoted apoptosis (Fig 4A). This finding was verified by TUNEL staining of tsc2^-/-^ MEF cells showing that inhibition of both mTORC1 and TGM2 promoted cell death three-fold compared with the vehicle control and treatment with either rapamycin or KCC009 alone (Fig 4B). These data further prove our hypothesis that the compensatory increase in TGM2 could be responsible for rapamycin resistance and that additionally targeting TGM2 might aid rapamycin therapy.

### Rapamycin-induced TGM2 increase is autophagy-dependent

**Fig 5:**
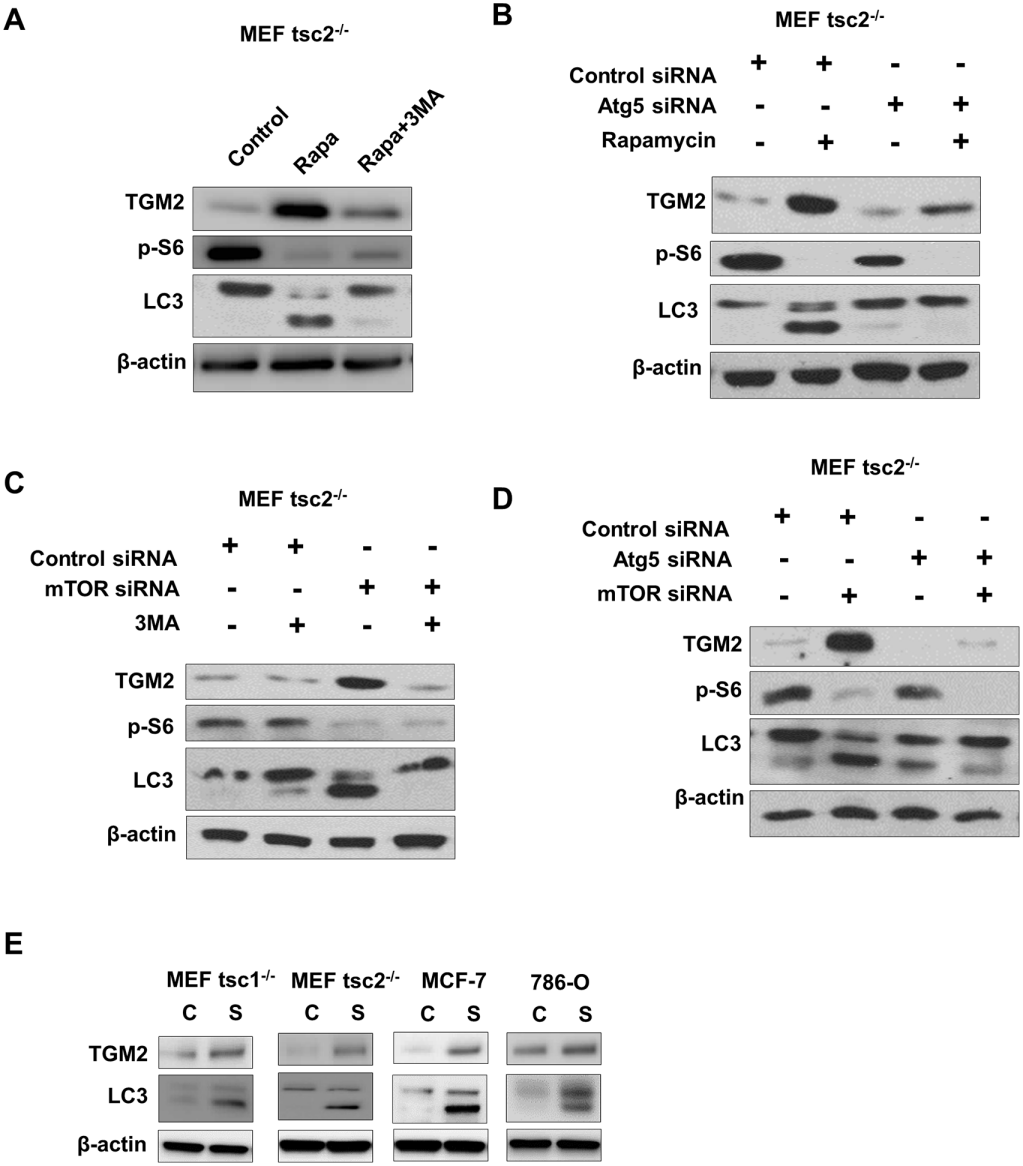
Rapamycin treatment-induced increased TGM2 is autophagy dependent. (A) Immunoblots for TGM2, phospho-S6 and LC3 in vehicle-, rapamycin- and rapamycin plus 3MA 33MA-treated Tsc2^-/-^ MEF cells. (B) Immunoblots for TGM2, phospho-S6 and LC3 in control siRNA-, Atg5 siRNA-, and Atg5 siRNA and rapamycin-treated Tsc2^-/-^ MEF cells. (C) Immunoblots for TGM2, phospho-S6 and LC3 in control siRNA-, mTOR siRNA-, and mTOR siRNA and 3MA-treated Tsc2^-/-^ MEF cells. (D) Immunoblots for TGM2, phospho-S6 and LC3 in control siRNA-, mTOR siRNA-, and mTOR and Atg5 siRNA-treated Tsc2^-/-^ MEF cells. (E) Immunoblots for TGM2 and LC3 in full medium (10% FBS) or starvation (FBS free) condition in Tsc1^-/-^ and Tsc2^-/-^ MEFs, MCF-7 and 786-O cells.

Given that mTORC1 signaling inhibits the known autophagy pathway, rapamycin could promote cancer cell autophagy [16]. To understand whether rapamycin-triggered autophagy is responsible for increased TGM2, we assessed the autophagy status of tsc2^-/-^ MEF cells in the presence of rapamycin. We found that rapamycin could promote the conversion of LC3-I to LC3-II and that the autophagy-specific inhibitor 3-Methyladenine (3MA) inhibited this conversion. Elevated TGM2 levels are attenuated by the 3MA due to reduced autophagy (Fig 5A). We further analyzed TGM2 expression by knocking down the autophagy-specific gene Atg5. Loss of Atg5 reduced rapamycin-triggered TGM2 expression (Fig 5B). 3MA and Atg5-knockdown inhibited mTORC1 inhibition-triggered TGM2 expression (Fig 5C and 5D). Additionally, to test whether induction of TGM2 depends on autophagy, we starved the cells to induced autophagy and observed the TGM2 expression in Tsc1^-/-^ and Tsc2^-/-^ MEFs, MCF-7 and 786-O cells. It showed that autophagy caused by starvation also could induce TGM2 expression (Fig 5E). Our data suggest that rapamycin-induced autophagy might contribute to the compensatory increase in TGM2 in mTORC1-hyperactive cells.

### Dual inhibition of mTORC1 and TGM2 reverses xenograft tumor development

**Fig 6:**
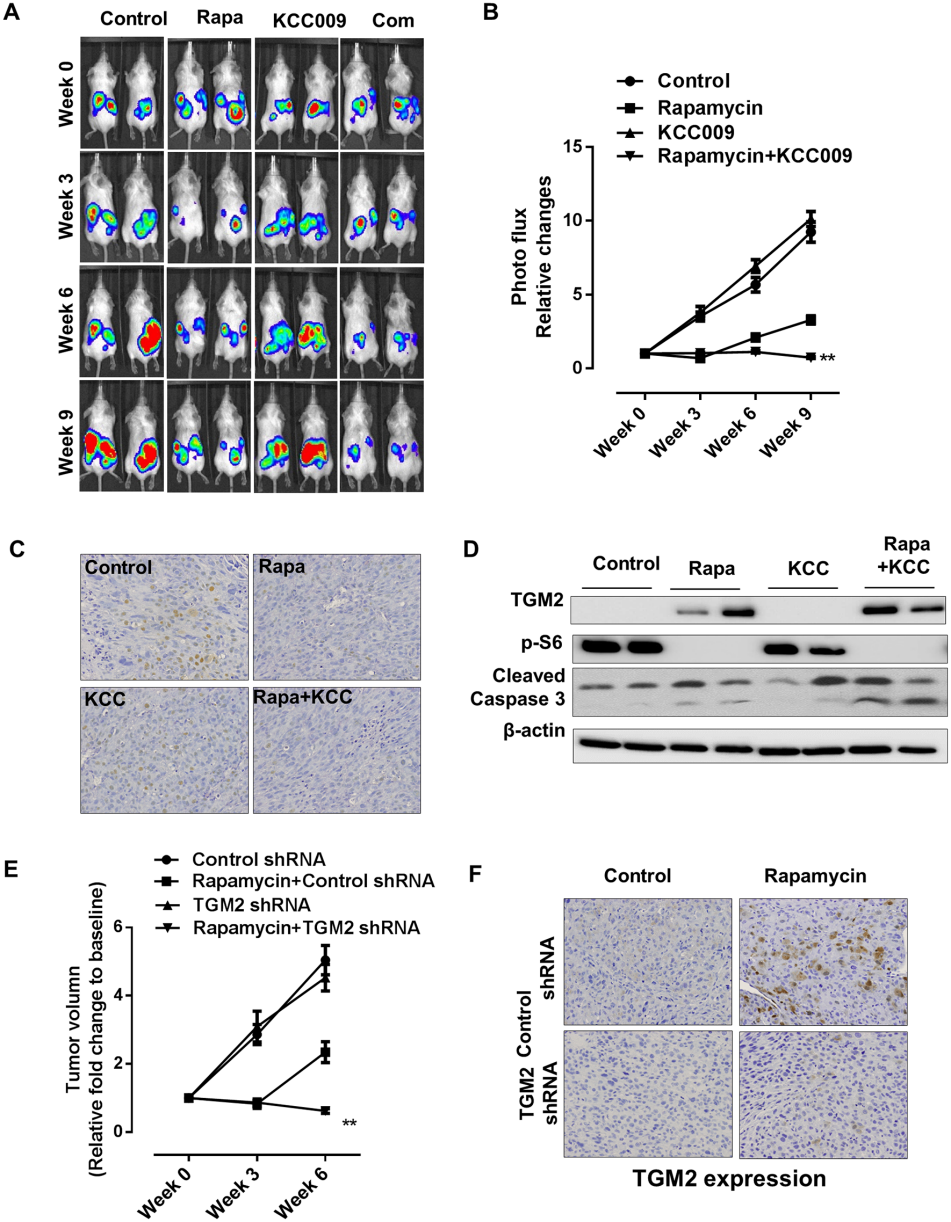
Dual inhibition of mTORC1 and TGM2 causes xenograft tumor regression. Female CB17-scid mice were s.c. inoculated with luciferase-labeled MCF-7 cells. Mice were treated with vehicle, rapamycin, KCC009, or rapamycin and KCC009 for 9 weeks. (A and B) Representative images of mice bearing MCF-7 xenograft tumors treated with vehicle control, rapamycin, KCC009, or rapamycin and KCC009. Xenograft tumor bioluminescent intensity was recorded and quantified every three weeks. The y-axis indicates relative tumor growth vs. the baseline quantification before drug treatment (n = 8). (C) Representative images of immunohistochemical staining for cell proliferation marker PCNA in tumors from mice treated with vehicle, rapamycin, KCC009, or rapamycin and KCC009. (D) Immunoblot of tumor lysates for TGM2, p-S6, and cleaved caspase 3 in tumors from mice treated with vehicle, rapamycin, KCC009, or rapamycin and KCC009. (E) Mice were inoculated with control or TGM2 shRNA knockdown stable MCF-7 cell lines. Vehicle control or rapamycin was applied to mice with tumor burden. Tumor volume (relative fold change to baseline) was recorded and quantified every three weeks until 6 weeks. (F) Representative images of immunohistochemical staining for TGM2 in tumors from mice inoculated with control or TGM2 shRNA knockdown stable MCF-7 cell lines treated with vehicle or rapamycin. All data are shown as means ± S.D. ** P < 0.01, *P < 0.05, Student’s t-test.

To reconcile the efficacy of combining mTORC1 and TGM2 inhibition in tumor cells with mTORC1-hyperactive cells *in vivo*, we developed an MCF-7-luciferase-tagged xenograft tumor model. Mice bearing MCF-7-luciferase-tagged xenograft tumors were treated with rapamycin and KCC009 either singly or in combination, and tumor growth was monitored at various time points during treatment by non-invasive imaging using an IVIS platform. Compared with the vehicle control, rapamycin reduced tumor growth capacity while KCC009 did not affect tumor growth. In contrast, treatment with both rapamycin and KCC009 for six and nine weeks post-inoculation fully suppressed xenograft tumor progression. Most importantly, the combined treatment resulted in tumor regression (Fig 6A). This benefit was also evidenced by statistical analysis of photon flux in response to combination treatment (Fig 6B). Immunohistochemical staining revealed that the combined rapamycin and KCC009 treatment reduced the cell-proliferation marker proliferating cell nuclear antigen (PCNA), suggesting reduced tumor compared with the single treatments (Fig 6C). Moreover, western blot analysis revealed increased levels of an indicator of apoptosis, cleaved caspase 3, in response to the combined treatment compared with the single-agent treatments (Fig 6D). To further validate that blocking TGM2 could benefit mTORC1 inhibitors treatment, we performed xenograft model using TGM2 knockdown MCF-7 cell line instead of KCC009 treatment. It showed that dual targeting TGM2 and mTORC1 for six weeks post-inoculation caused regression (Fig 6E). We also performed TGM2 protein immunohistochemical staining in xenograft tumors. Rapamycin promotes expression level of TGM2 in the tumors of mice inoculated with the control shRNA MCF-7 cell line. However, TGM2 shRNA could fully suppress the expression level of TGM2 in tumors treated with rapamycin (Fig 6F).

## Discussion

Mammalian target of rapamycin (mTOR) is a serine/threonine protein kinase that plays crucial roles in transcriptional regulation, initiation of protein synthesis, ribosome biogenesis, metabolism and apoptosis. Aberrant mTORC1 activation is frequently observed in cancers and other diseases resulting from mutations in numerous oncogenes and tumor suppressors [17, 18]. The mTOR signaling pathway has become a compelling target for cancer treatment [19–21]. Although mTOR inhibitors are active against some cancer types, only a small fraction of patients treated with these agents exhibit substantial clinical benefits [22]. Several studies have shown that mTORC1 inhibitors are cytostatic rather than cytotoxic [23]; consequently, the emergence of drug resistance may ultimately limit the use of mTOR inhibitor therapy and generates an immediate need for the identification of new determinants of cell sensitivity to mTORC1 inhibition. Recently, many studies has shown several mechanisms associated with rapamycin resistance, which including promyelocytic leukemia (PML) gene [24], JAK2/STAT5 [25], Notch1 [26] and MAPK [27] mediated resistance to mTOR-targeted therapies in cancer. However, underpinning mechanism of mTORC1 inhibitor resistance remains obscure. Here, we took a bioinformatics approach focused on the observation that rapamycin promoted TGM2 expression in mTORC1-hyperactive cells. We have demonstrated a compensatory increase in TGM2 in response to rapamycin treatment in various mTORC1-hyperactive cancer cells due to various tumor suppressor mutations. More interestingly, TGM2 blockade sensitizes mTORC1-hyperactive cancer cells to rapamycin treatment.

Several studies have shown that tissue transglutaminase, also known as transglutaminase 2, is involved in tumor drug resistance and evasion of apoptosis [28]. TGM2 functions to block apoptosis through various mechanisms involving both GTP-binding activities and transamidation. TGM2 also promotes cell survival that depends on aberrant NF-κB activity [29–34]. Moreover, TGM2-mediated transamidation of RB in the nucleus protects RB protein from degradation to promote survival [35], and the association of TGM2 with some integrin family members promotes cell anchoring to the ECM, activating cell survival pathways [36–38]. In several studies, a TGM2-induced epithelial–mesenchymal transition (EMT) was reported to be responsible for drug resistance [39, 40]. TGM2-mediated chemoresistance was shown to regulate ECM proteins for tumor cell survival [37]. More interestingly, TGM2 could active PI3K/mTORC1 signaling pathway to be implicated in various aspects of cancer progression including cell survival and chemo-resistance [41, 42]. Taken together, increasing lines of evidence have indicated a crucial role for TGM2 in resistance to numerous drugs [28]. Consequently, TGM2 targeting has become a potential anticancer strategy [43–45], and an increasing number of distinct TGM2 inhibitors have been developed.

Herein, we found that rapamycin treatment promotes TGM2 expression in mTORC1-hyperactive cancer cells, including tsc1-/- and tsc2-/- MEFs, TSC2-deficient patient-derived cells, MCF-7 and 786-O cells. The rapamycin-triggered increase in TGM2 might be autophagy-dependent. Co-targeting of mTORC1 and TGM2 reduced cell viability and promoted apoptosis. The benefits of combined treatment were also evaluated in an MCF-7 xenograft tumor model. TGM2-triggered pro-survival events including NF-κB activation will lead us to an understanding of rapamycin resistance in mTORC1-hyperactive cancer cells. In contrast, Zha et al. reported that rapamycin could reduce NF-κB signaling pathway activity in tsc2-/- MEF cells [46]. TGM2 activation-driven resistance of rapamycin-treated cells is independent of the NF-κB signaling pathway. Mikaelian et al. reported that genetic and pharmacologic inhibition of mTORC1 promoted EMT in breast cancer cell lines [47]; this result is consistent with TGM2-induced EMT, which could explain the rapamycin resistance of mTORC1-hyperactive cancer cells. Further study downstream of TGM2 in rapamycin-resistant cancer cells is urgently needed to establish an understanding of the cytostatic mechanism of rapamycin.

To understand why TGM2 up-regulation would occur then blocking mTORC1 with rapamycin, our data suggest that rapamycin-induced autophagy might contribute to the compensatory increase in TGM2 in mTORC1-hyperactive cells. However, the underlying mechanism is unclear. There have been suggestions in the literature that NF-κB could regulate expression of TGM2 by activation of transcription through interaction of p65 with two independent consensus NF-κB binding sites within the TGM2 promoter [48, 49]. Furthermore, in TSC2^-/-^ MEFs, the rapamycin-mediated inhibition of deregulated mTOR activity restored NF-κB activation [50]. This is consistent with TGM2 up-regulation induced by mTORC1 blockade through NF-κB activation. The mechanism that how autophagy induced by rapamycin regulates NF-κB signaling pathway activation in mTORC1-hyperactive cells need to be further investigated.

We have demonstrated a promising strategy of targeting both mTORC1 and TGM2 *in vitro* and in a xenograft tumor model, and we observed that this combined approach may have resulted in tumor regression. Further investigations are required to evaluate the therapeutic benefits in additional preclinical models of mTORC1-hyperactive tumors, including genetic models.

## Supporting Information

S1 Table Gene profile for bioinformatics analysis. (XLSX)
