# Expression of concern: Compensatory increase of transglutaminase 2 is responsible for resistance to mTOR inhibitor treatment

**DOI:** 10.1371/journal.pone.0227851

**Published:** 2020-01-15

**Authors:** The PLOS ONE Editors

After publication of this article [1], the authors notified the journal office of concerns about the reliability of results presented in Fig 6. They had attempted to replicate the *in vivo* tumor results, and results of these post-publication experiments did not support the original findings that combinatorial treatment with Rapamycin and KCC009 had a greater effect than Rapamycin alone (Supporting Information S1 and S2 Files).

The authors subsequently noted that they had found evidence of mycoplasma contamination in the MCF-7-Luciferase cell line used in the initial post-publication replication experiments. They repeated the experiments using a fresh vial of MCF-7 cells, although different methods were used (e.g. chemical versus shRNA inhibition) and outcome data were collected over a shorter timecourse (4 weeks in the replication experiments versus 6 or 9 weeks in the original experiments). The results of these experiments are reported here in an updated version of Fig 6. The Methods used for the replication experiments are included below.

**Fig 6:**
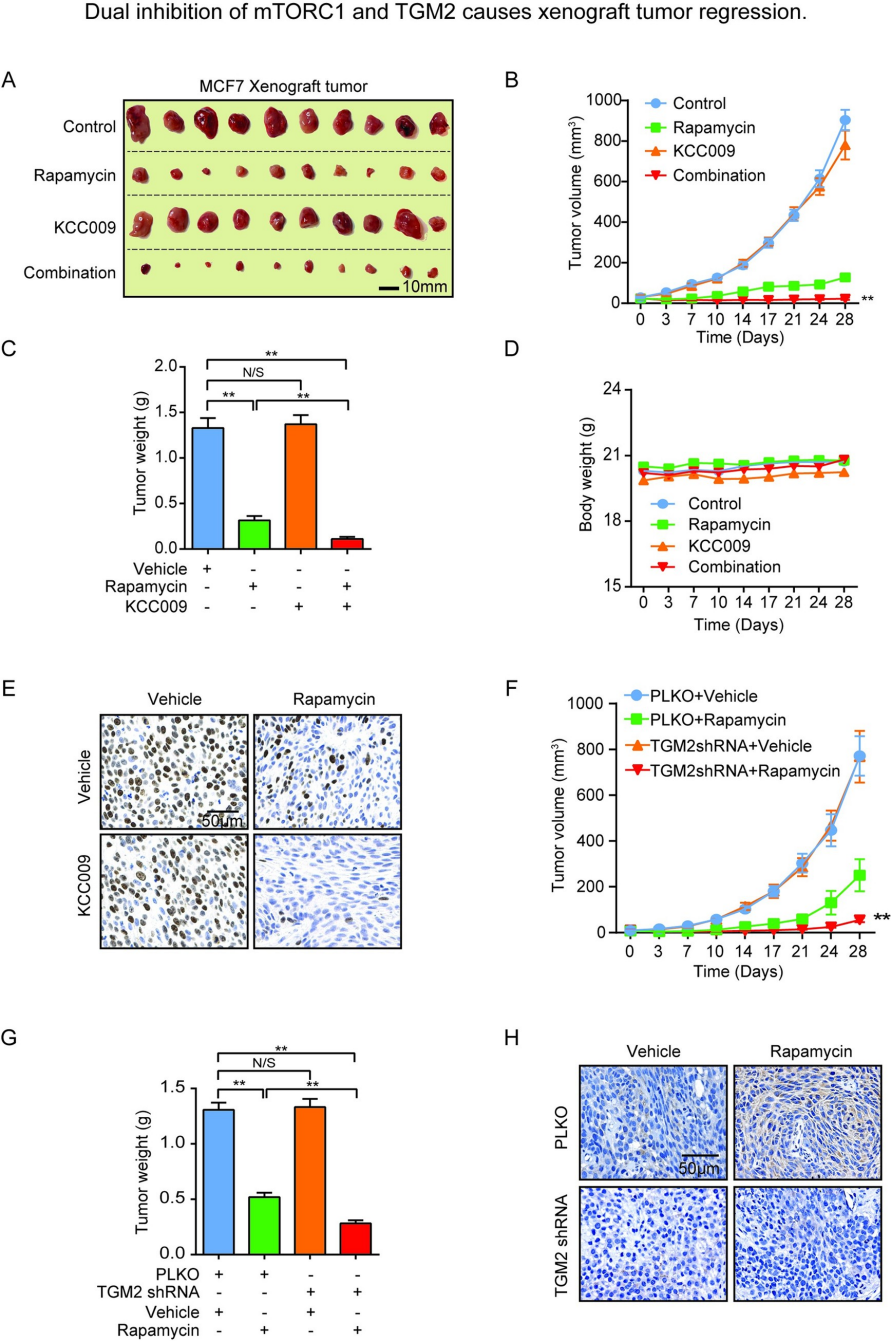
Dual inhibition of mTORC1 and TGM2 causes xenograft tumor arrest. (A) Representative image and (B) Tumor growth and (C) Tumor weight of MCF-7 xenograft tumors treated with vehicle control, rapamycin, KCC009, or combination of rapamycin and KCC009 (n = 10). (D) Body weight of the mice with tumor burden of MCF-7 cells. The mice were treated as indicated compounds or vehicle control (n = 5). (E) Representative images of immunohistochemical staining for cell proliferation marker PCNA in tumors from mice treated with vehicle, rapamycin, KCC009, or combination of rapamycin and KCC009. (F) Tumor growth and (G) Tumor weight of MCF-7 xenograft tumor with PLKO or TGM2 shRNA knockdown stable MCF-7 cell lines. Vehicle control or rapamycin was applied to mice with tumor burden (n = 10). (H) Representative images of immunohistochemical staining for TGM2 in tumors from mice inoculated with PLKO or TGM2 shRNA knockdown stable MCF-7 cell lines treated with vehicle control or rapamycin. All data are shown as means ± S.E.M ** P < 0.01, *P < 0.05, Student’s t-test.

The authors summarized the results of the Fig 6 replication experiments as follows:

To reconcile the efficacy of combining mTORC1 and TGM2 inhibition in tumor cells with mTORC1-hyperactive cells in vivo, an MCF-7 xenograft tumor model was used. Mice bearing MCF-7 xenograft tumors were treated with rapamycin and KCC009 either singly or in combination, and tumor growth was monitored at various time points during treatment by caliper measurement. Compared with the vehicle control, rapamycin reduced tumor growth capacity while KCC009 did not affect tumor growth. In contrast, treatment with both rapamycin and KCC009 fully suppressed xenograft tumor progression (new **Fig 6A**). This benefit was also evidenced by statistical analysis of tumor volume and weight in response to combination treatment (new **Fig 6B–6C**). The combinational treatment was shown not to have toxic effect according to body weight monitoring (new **Fig 6D**). Immunohistochemical staining revealed that the combined rapamycin and KCC009 treatment reduced the cell-proliferation marker proliferating cell nuclear antigen (PCNA), suggesting reduced tumor compared with the single treatments (new **Fig 6E**). To further validate that blocking TGM2 could benefit mTORC1 inhibitors treatment, we performed xenograft model using TGM2 knockdown MCF-7 cell line instead of KCC009 treatment. It showed that dual targeting TGM2 and mTORC1 for four weeks post-inoculation caused tumor arrest by analysis of tumor volume and weight (new **Fig 6F and 6G**). We also performed TGM2 protein immunohistochemical staining in xenograft tumors. Rapamycin promotes expression level of TGM2 in the tumors of mice inoculated with the control shRNA MCF-7 cell line. However, TGM2 shRNA could fully suppress the expression level of TGM2 in tumors treated with rapamycin (new **Fig 6H**).

A member of *PLOS ONE*’s Editorial Board advised that the results of these experiments support the conclusions reported in [1].

In addition, a reader raised concerns that the lower panels of Fig 3B (TGM2 shRNA, Control and Rapamycin panels) in [1] appear to duplicate the left panels of Fig 1F (Control, Vehicle and H_2_O_2_ panels, respectively) in [2]. The authors noted that this was due to an error in preparing the *PLOS ONE* figure, and provide here an updated version of Fig 3 in which all four panels have been replaced with data from a replication experiment. The raw image data from the original experiment and the replication experiment are in S4 File. As the duplicated panels are not licensed for distribution under the terms of the Creative Commons Attribution License, this content (original Fig 3B TGM2 shRNA, Control and Rapamycin panels in [1]) has been removed from the original version of the *PLOS ONE* article.

**Fig 3:**
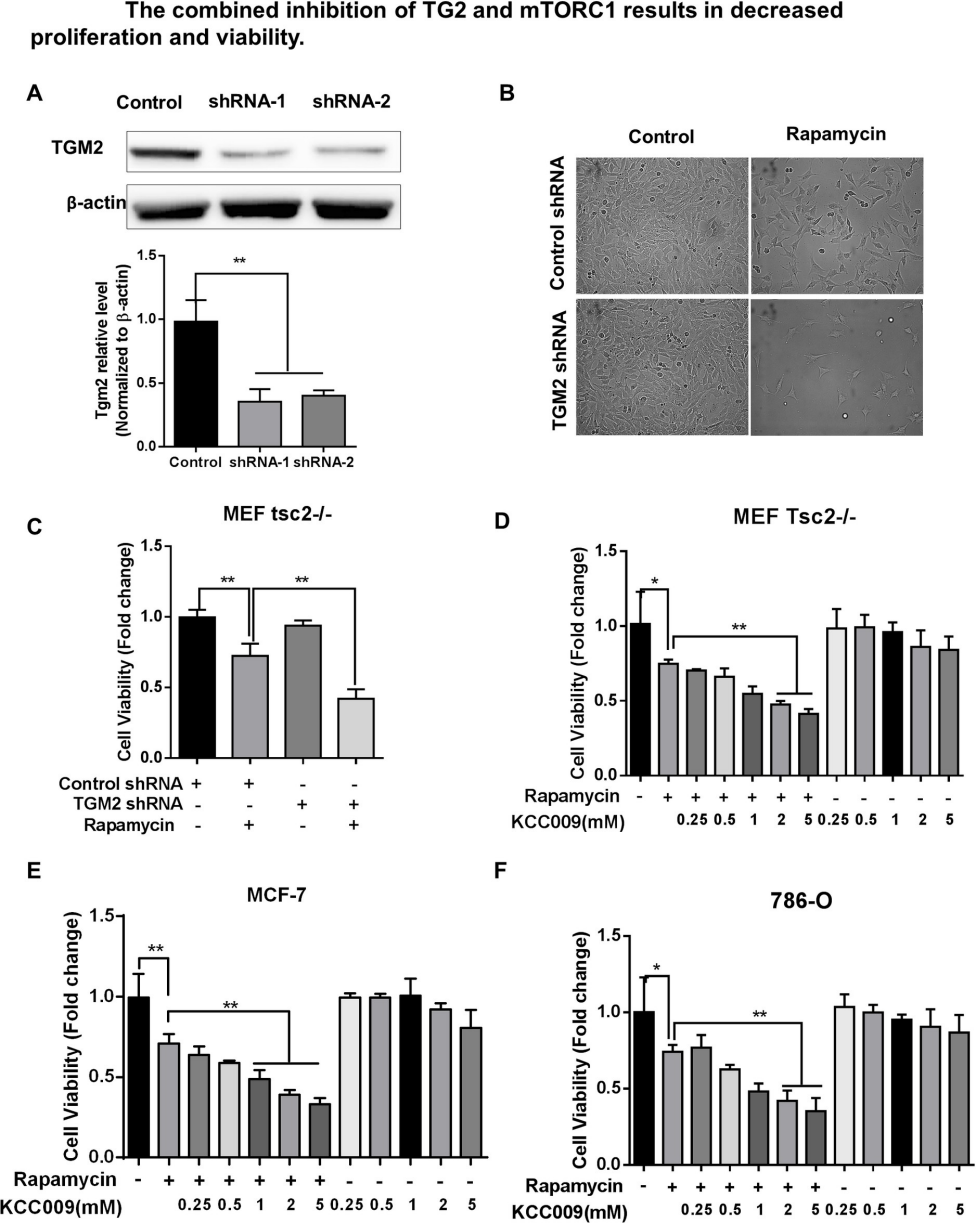
Combined TGM2 and mTORC1 inhibition leads to morphological changes and reduced viability. (A) Tsc2^-/-^ MEF cells were treated with siRNAs against TGM2 and lysates were used for immunoblotting. Densitometry is shown in the bar chart (n = 3). (B) Tsc2^-/-^ MEF cells were treated with either TGM2 siRNA or 20 nM rapamycin for 24 hours and cell morphologies were recorded using phase-contract microscopy. (C) Tsc2^-/-^ MEF cell viability following treatment with TGM2 siRNA and 20 nM rapamycin (n = 8). (D) Tsc2^-/-^ MEF cell viability following treatment with 20 nM rapamycin and the indicated doses of TGM2 inhibitor KCC009. (E) Viability of MCF-7 cells treated with 20 nM rapamycin and the indicated doses of TGM2 inhibitor KCC009 (n = 8). (F) Viability of 786-O cells treated with 20 nM rapamycin and the indicated doses of TGM2 inhibitor KCC009 (n = 8). All data are shown as means ± S.D. ** P < 0.01, *P < 0.05, Student’s t-test.

The primary data underlying results in this article were not included with the published article although the Data Availability Statement for this article stated, “All relevant data are within the paper and its Supporting Information files.” The authors provide in S1–S8 Files the underlying data supporting results reported in [1] and in this notice.

In addition, a reader questioned the origin of results reported in the original published versions of Figs 1B and 6A. We attempted to contact the corresponding author’s institution about this issue but to date this remains unresolved.

The *PLOS ONE* Editors issue this Expression of Concern to notify readers of the above concerns and relay the supporting data provided by authors post-publication.

## Methods for replication studies

1 x 10^6^ MCF-7 cells with a luciferase tag were inoculated in both flanks of mice. Four weeks post inoculation, mice bearing subcutaneous tumors were randomized into four groups: vehicle control (n = 5; 10% DMSO i.p.), rapamycin (n = 5; 1 mg/kg/day i.p.), KCC009 (n = 5; 50 mg/kg/day in 10% DMSO i.p.) and rapamycin plus KCC009 (n = 5; 1 mg/kg/day and 50 mg/kg/day, respectively, i.p.). Drug treatments were initiated four weeks post-inoculation, and tumor growth was monitored by tumor diameters (L and W) using caliper for six weeks. Tumor volume was calculated using the formula V = (W^2^ × L)/2. MCF7 cells with a luciferase tag were tested for mycoplasma using PCR. In brief, 100 μl of cell culture supernatant from a dense culture (80–100% confluent) were taken into a 1.5ml tube. Heat the sample for 5 min at 95°C and spin it for 2 min in a bench centrifuge at maximum speed. Tag polymerase was used to amplify the mycoplasma DNA. Primers were shown below: Forward 5’CGCCTGAGTAGTACGTTCGC3’, Reverse 5’GCGGTGTGTACAAGACCCGA3’. Further test for mycoplasma was done using LONZA MycoAlert^TM^ Mycoplasma Detection Kit followed by manufacture’s instruction.

A fresh vial of mycoplasma free MCF7 cells (1 x 10^6^) were inoculated into both flanks of mice. Two weeks post inoculation, mice bearing subcutaneous tumors were randomized into four groups: vehicle control (n = 5; 10% DMSO i.p.), rapamycin (n = 5; 1 mg/kg/day i.p.), KCC009 (n = 5; 50 mg/kg/day in 10% DMSO i.p.) and rapamycin plus KCC009 (n = 5; 1 mg/kg/day and 50 mg/kg/day, respectively, i.p.). Drug treatments were initiated two weeks post-inoculation, and tumor growth was monitored by tumor diameters (L and W) using caliper for four weeks. Tumor volume was calculated using the formula V = (W^2^ × L)/2.

## Supporting information

S1 File Tumor growth data I. (PZF)

S2 File Tumor growth data II. (XLSX)

S3 File Underlying data for replication experiments reported in the updated version of Fig 6. Original image files for panel E and H, individual-level quantitative data to support all graphs, and information as to time points at which results in panel A,C,E,G and H were collected. All tumor samples were collected at week 4. The authors noted that the new tumor cell line grew faster compared with the one used for experiments reported in the original Fig 6. At Week 4, all tumors almost reached the limitation which allowed in the animal facility.
(ZIP)

S4 File Underlying image data for the original experiment shown in Fig 3B [1] and for the replication experiment shown in the updated version of this figure. (ZIP)

S5 File Raw flow cytometry data supporting results reported in [1]. (ZIP)

S6 File Blot image data supporting Figs 1G, 2B, 2D, 2F and 2G. (PDF)

S7 File Blot image data supporting Figs 3A and 5. (PDF)

S8 File Raw data underlying the quantitative results reported in Figs 1, 2, 3, 4 and S1 File. (ZIP)
