# Supplementary figures and images for: Expression of concern: Compensatory increase of transglutaminase 2 is responsible for resistance to mTOR inhibitor treatment
Source: PLoS One. 2020 Jan 15;15(1):e0227851. doi: 10.1371/journal.pone.0227851 (PMC6961868; doi:10.1371/journal.pone.0227851)

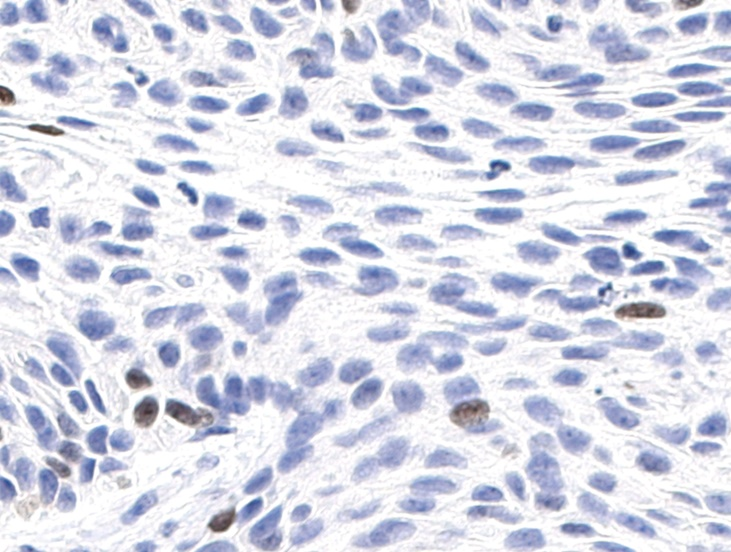

Supplement: S3 File — Original image files for panel E and H, individual-level quantitative data to support all graphs, and information as to time points at which results in panel A,C,E,G and H were collected. All tumor samples were collected at week 4. The authors noted that the new tumor cell line grew faster compared with the one used for experiments reported in the original Fig 6. At Week 4, all tumors almost reached the limitation which allowed in the animal facility. (ZIP) [file pone.0227851.s003.zip › Original data for editor/Panel E and H original images/PCNA/Com-zoom used for new Figure 6.tif]

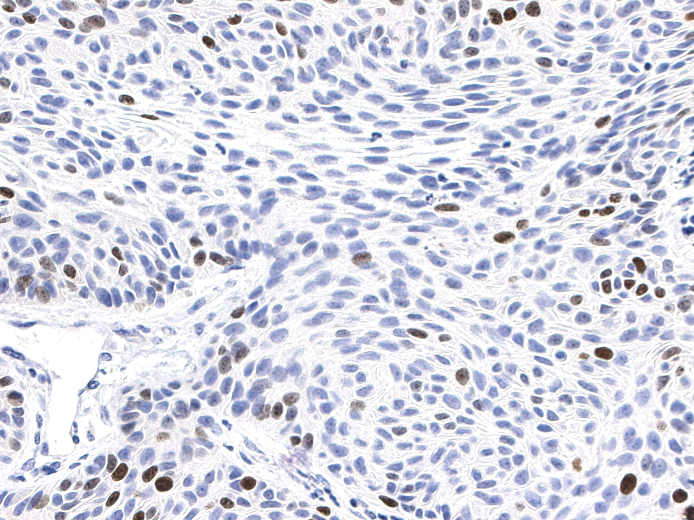

Supplement: S3 File — Original image files for panel E and H, individual-level quantitative data to support all graphs, and information as to time points at which results in panel A,C,E,G and H were collected. All tumor samples were collected at week 4. The authors noted that the new tumor cell line grew faster compared with the one used for experiments reported in the original Fig 6. At Week 4, all tumors almost reached the limitation which allowed in the animal facility. (ZIP) [file pone.0227851.s003.zip › Original data for editor/Panel E and H original images/PCNA/Com.tif]

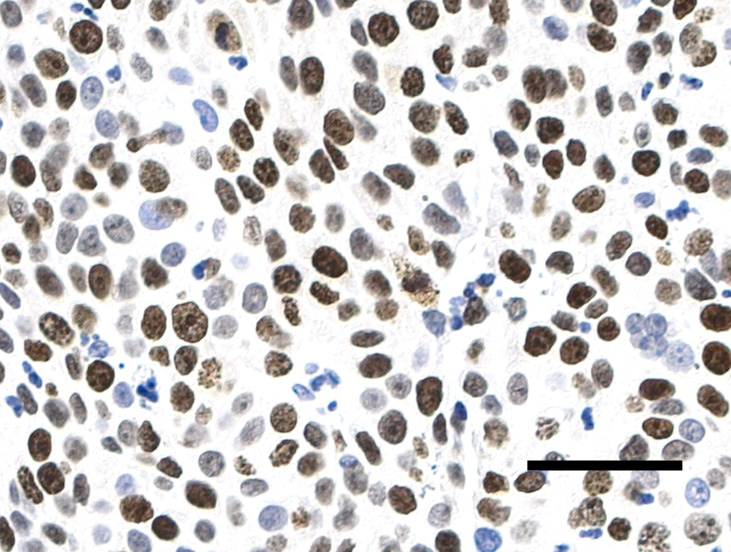

Supplement: S3 File — Original image files for panel E and H, individual-level quantitative data to support all graphs, and information as to time points at which results in panel A,C,E,G and H were collected. All tumor samples were collected at week 4. The authors noted that the new tumor cell line grew faster compared with the one used for experiments reported in the original Fig 6. At Week 4, all tumors almost reached the limitation which allowed in the animal facility. (ZIP) [file pone.0227851.s003.zip › Original data for editor/Panel E and H original images/PCNA/Control-zoom used for new Figure 6.tif]

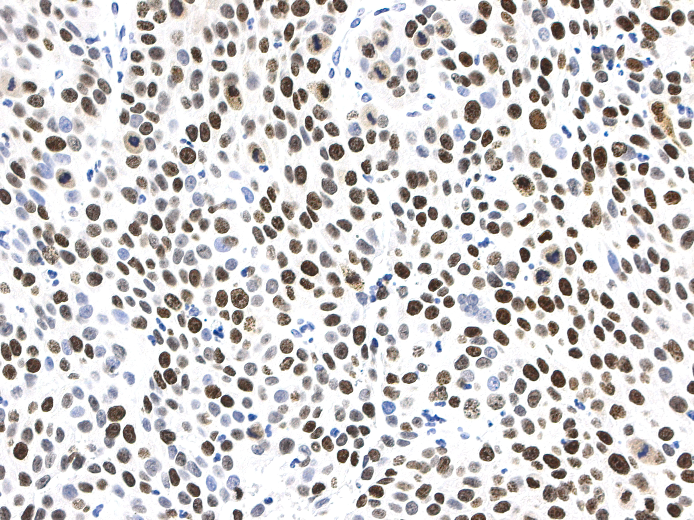

Supplement: S3 File — Original image files for panel E and H, individual-level quantitative data to support all graphs, and information as to time points at which results in panel A,C,E,G and H were collected. All tumor samples were collected at week 4. The authors noted that the new tumor cell line grew faster compared with the one used for experiments reported in the original Fig 6. At Week 4, all tumors almost reached the limitation which allowed in the animal facility. (ZIP) [file pone.0227851.s003.zip › Original data for editor/Panel E and H original images/PCNA/Control.tif]

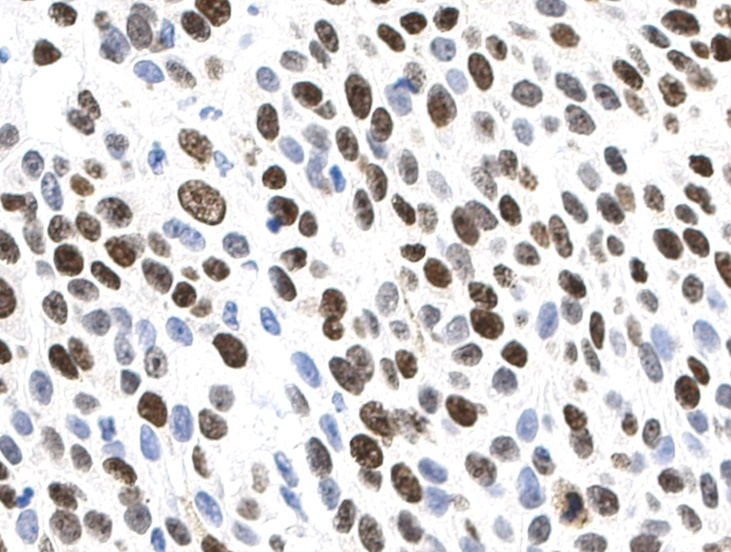

Supplement: S3 File — Original image files for panel E and H, individual-level quantitative data to support all graphs, and information as to time points at which results in panel A,C,E,G and H were collected. All tumor samples were collected at week 4. The authors noted that the new tumor cell line grew faster compared with the one used for experiments reported in the original Fig 6. At Week 4, all tumors almost reached the limitation which allowed in the animal facility. (ZIP) [file pone.0227851.s003.zip › Original data for editor/Panel E and H original images/PCNA/KCC009-zoom used for new Figure 6.tif]

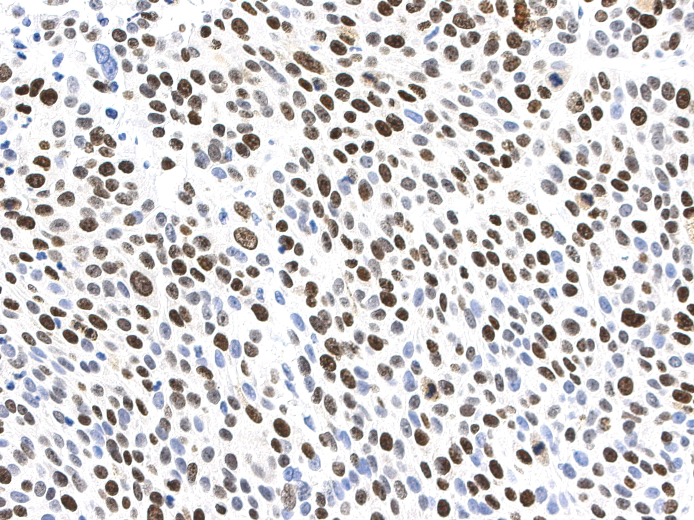

Supplement: S3 File — Original image files for panel E and H, individual-level quantitative data to support all graphs, and information as to time points at which results in panel A,C,E,G and H were collected. All tumor samples were collected at week 4. The authors noted that the new tumor cell line grew faster compared with the one used for experiments reported in the original Fig 6. At Week 4, all tumors almost reached the limitation which allowed in the animal facility. (ZIP) [file pone.0227851.s003.zip › Original data for editor/Panel E and H original images/PCNA/KCC009.tif]

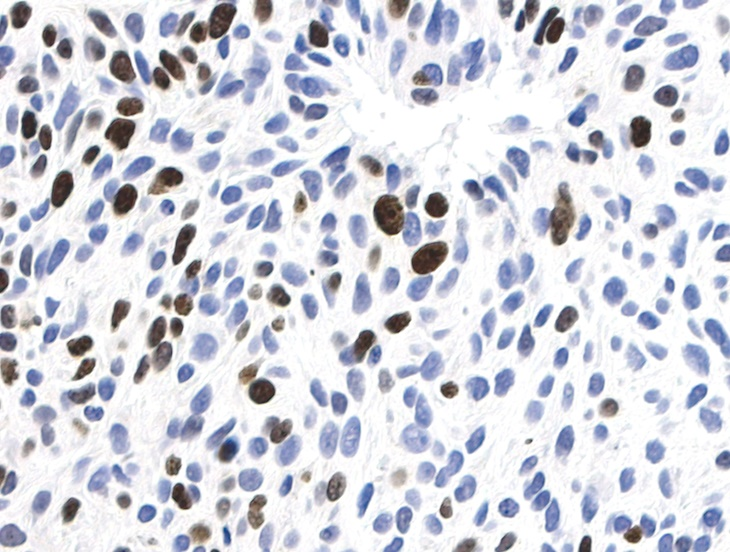

Supplement: S3 File — Original image files for panel E and H, individual-level quantitative data to support all graphs, and information as to time points at which results in panel A,C,E,G and H were collected. All tumor samples were collected at week 4. The authors noted that the new tumor cell line grew faster compared with the one used for experiments reported in the original Fig 6. At Week 4, all tumors almost reached the limitation which allowed in the animal facility. (ZIP) [file pone.0227851.s003.zip › Original data for editor/Panel E and H original images/PCNA/Rapa-zoom used for new Figure 6.tif]

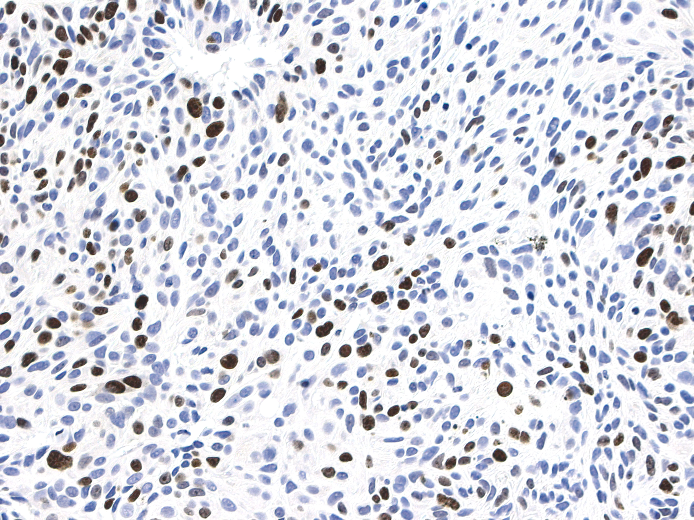

Supplement: S3 File — Original image files for panel E and H, individual-level quantitative data to support all graphs, and information as to time points at which results in panel A,C,E,G and H were collected. All tumor samples were collected at week 4. The authors noted that the new tumor cell line grew faster compared with the one used for experiments reported in the original Fig 6. At Week 4, all tumors almost reached the limitation which allowed in the animal facility. (ZIP) [file pone.0227851.s003.zip › Original data for editor/Panel E and H original images/PCNA/Rapamycin.tif]

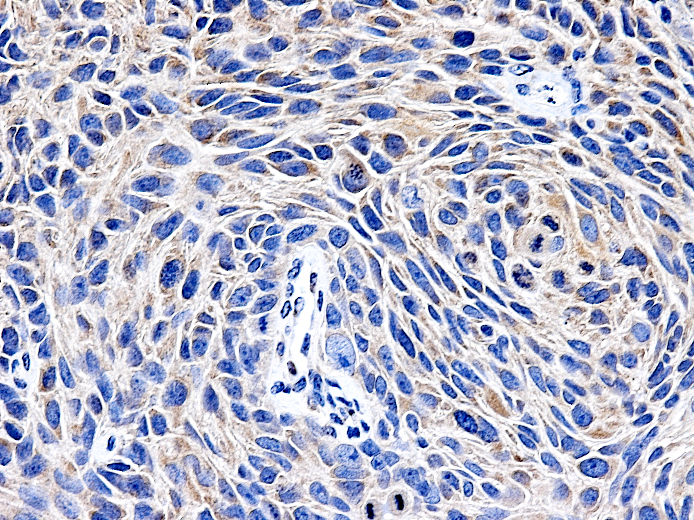

Supplement: S3 File — Original image files for panel E and H, individual-level quantitative data to support all graphs, and information as to time points at which results in panel A,C,E,G and H were collected. All tumor samples were collected at week 4. The authors noted that the new tumor cell line grew faster compared with the one used for experiments reported in the original Fig 6. At Week 4, all tumors almost reached the limitation which allowed in the animal facility. (ZIP) [file pone.0227851.s003.zip › Original data for editor/Panel E and H original images/TGM2/Rapamycin.tif]

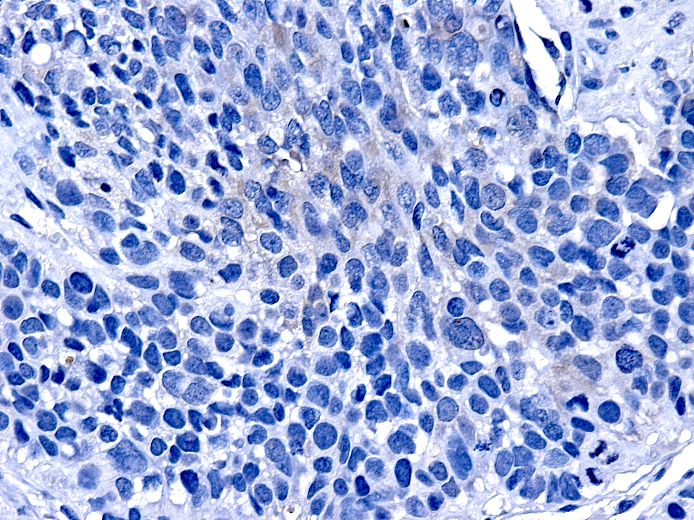

Supplement: S3 File — Original image files for panel E and H, individual-level quantitative data to support all graphs, and information as to time points at which results in panel A,C,E,G and H were collected. All tumor samples were collected at week 4. The authors noted that the new tumor cell line grew faster compared with the one used for experiments reported in the original Fig 6. At Week 4, all tumors almost reached the limitation which allowed in the animal facility. (ZIP) [file pone.0227851.s003.zip › Original data for editor/Panel E and H original images/TGM2/TGM shRNA+Rapamycin.tif]

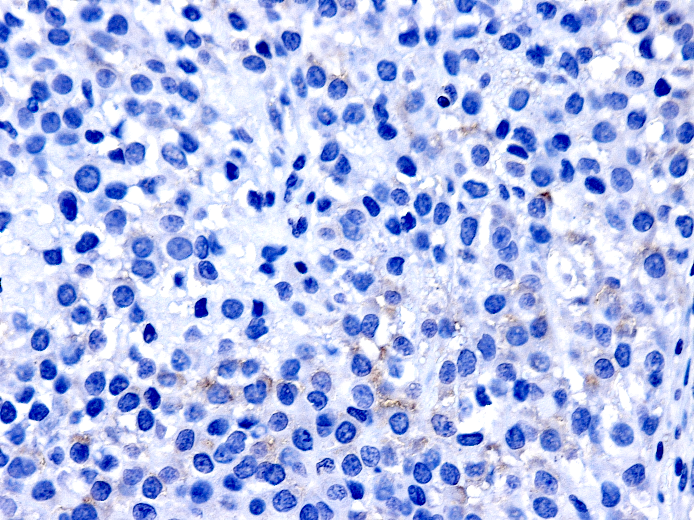

Supplement: S3 File — Original image files for panel E and H, individual-level quantitative data to support all graphs, and information as to time points at which results in panel A,C,E,G and H were collected. All tumor samples were collected at week 4. The authors noted that the new tumor cell line grew faster compared with the one used for experiments reported in the original Fig 6. At Week 4, all tumors almost reached the limitation which allowed in the animal facility. (ZIP) [file pone.0227851.s003.zip › Original data for editor/Panel E and H original images/TGM2/TGM shRNA.tif]

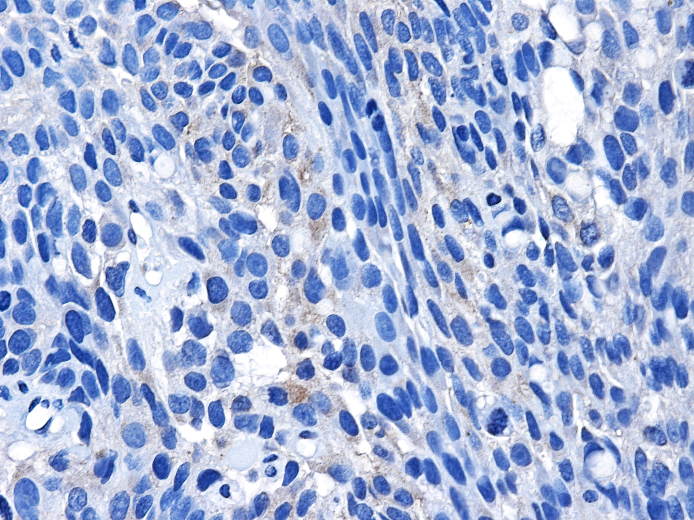

Supplement: S3 File — Original image files for panel E and H, individual-level quantitative data to support all graphs, and information as to time points at which results in panel A,C,E,G and H were collected. All tumor samples were collected at week 4. The authors noted that the new tumor cell line grew faster compared with the one used for experiments reported in the original Fig 6. At Week 4, all tumors almost reached the limitation which allowed in the animal facility. (ZIP) [file pone.0227851.s003.zip › Original data for editor/Panel E and H original images/TGM2/Vehicle.tif]

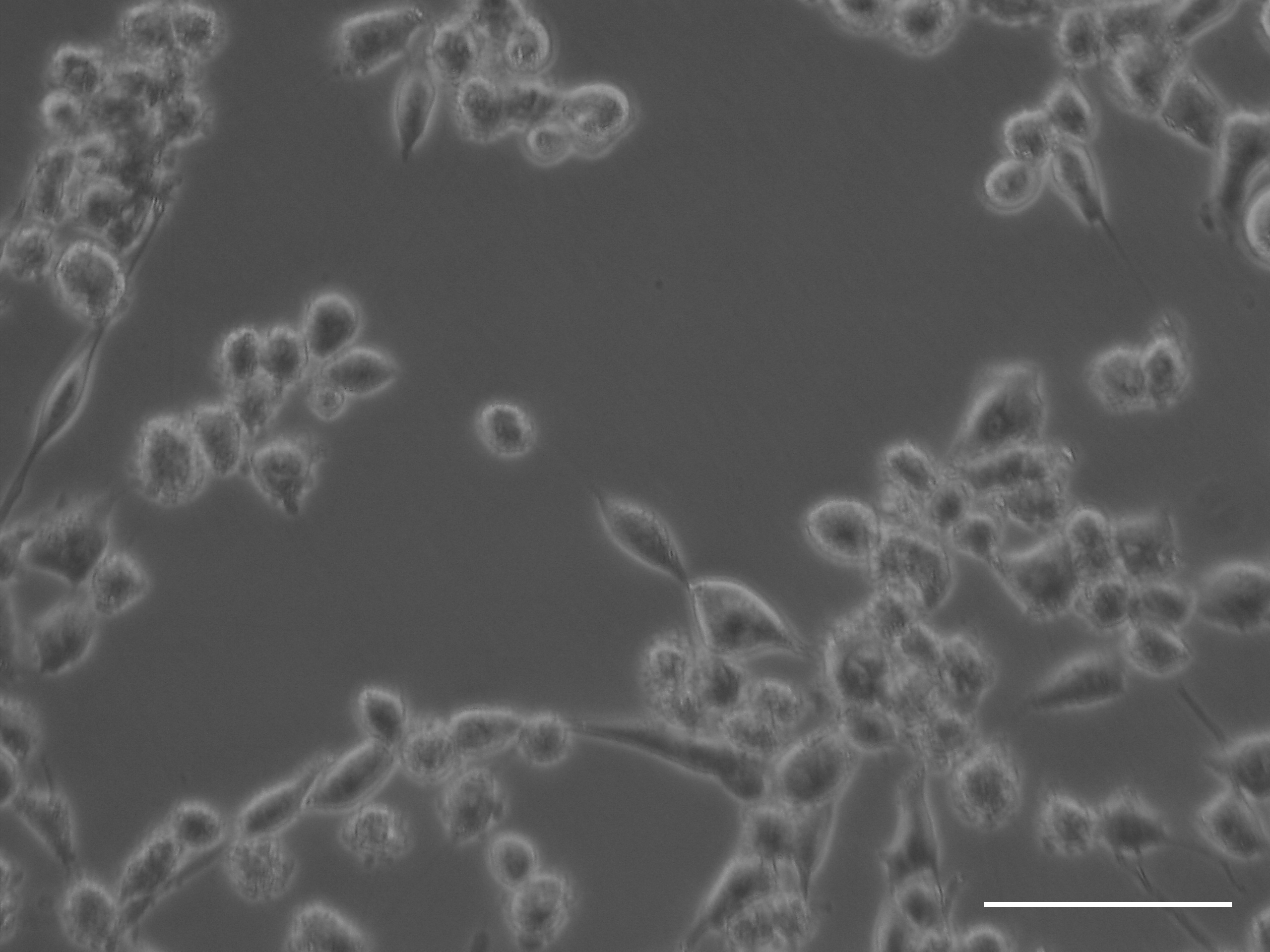

Supplement: S4 File — (ZIP) [file pone.0227851.s004.zip › S4-File_Raw Data and New Figure 3_Cell picture/1st version pictures/Combination.jpg]

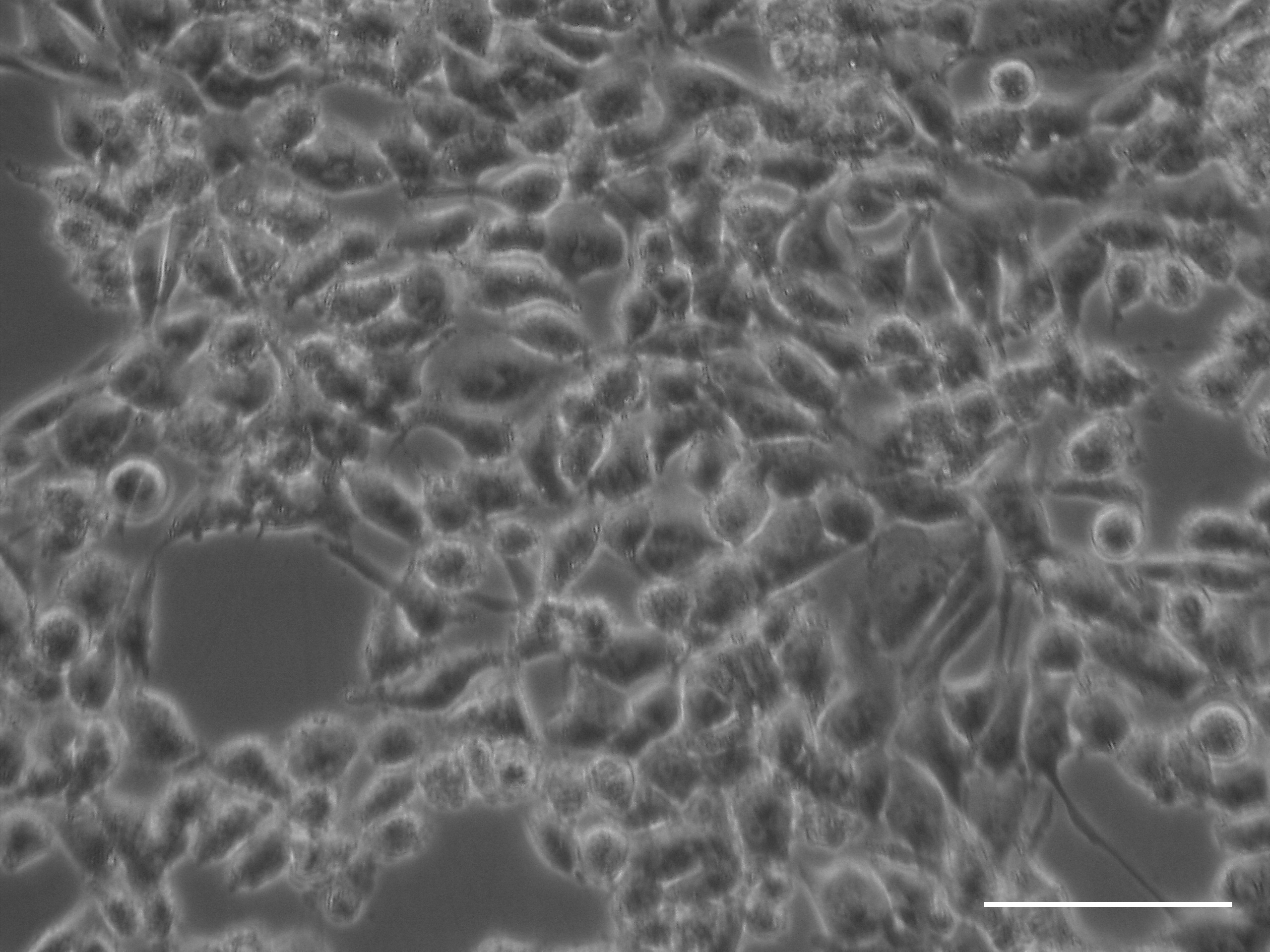

Supplement: S4 File — (ZIP) [file pone.0227851.s004.zip › S4-File_Raw Data and New Figure 3_Cell picture/1st version pictures/Control.jpg]

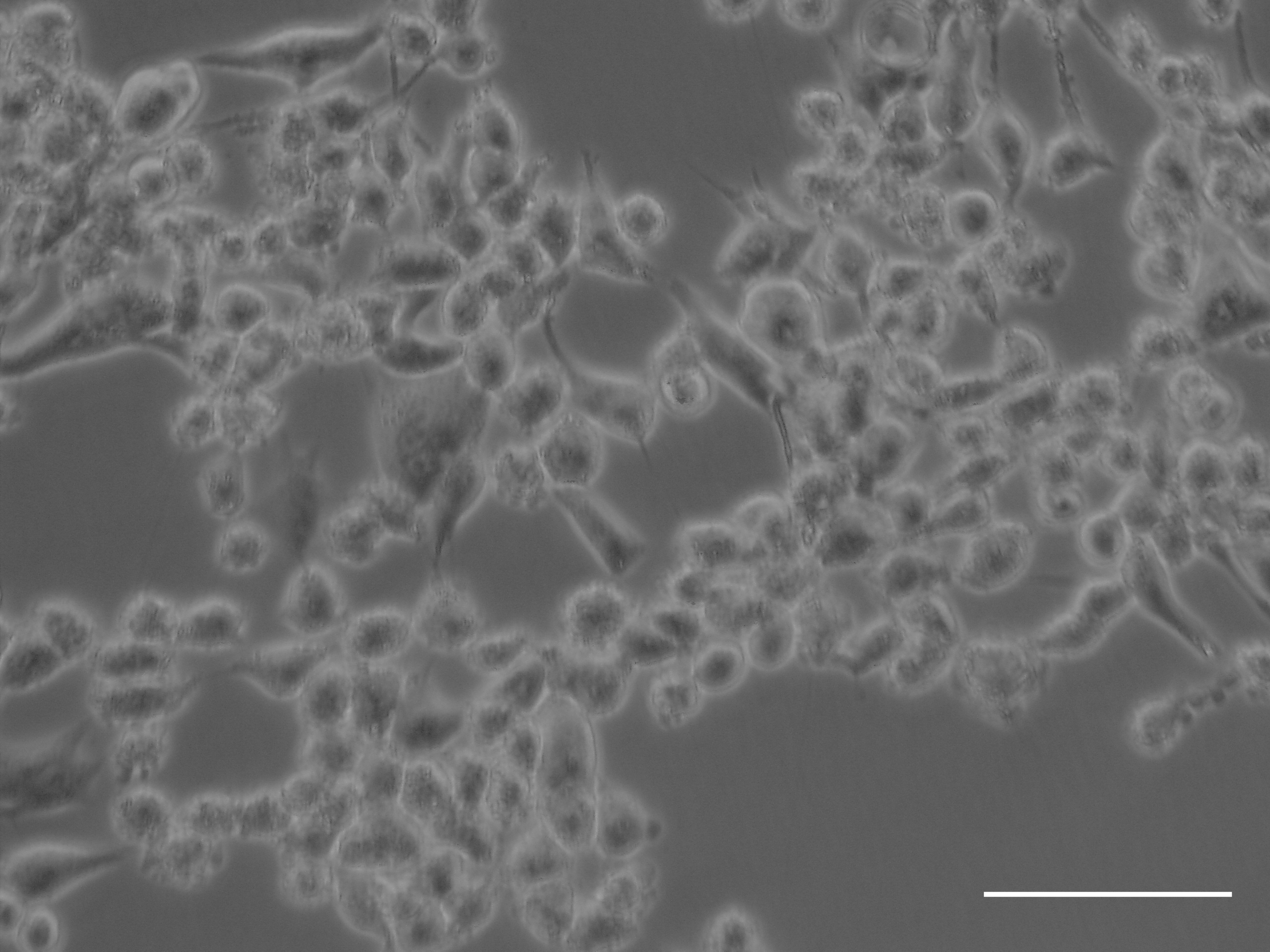

Supplement: S4 File — (ZIP) [file pone.0227851.s004.zip › S4-File_Raw Data and New Figure 3_Cell picture/1st version pictures/Rapamycin.jpg]

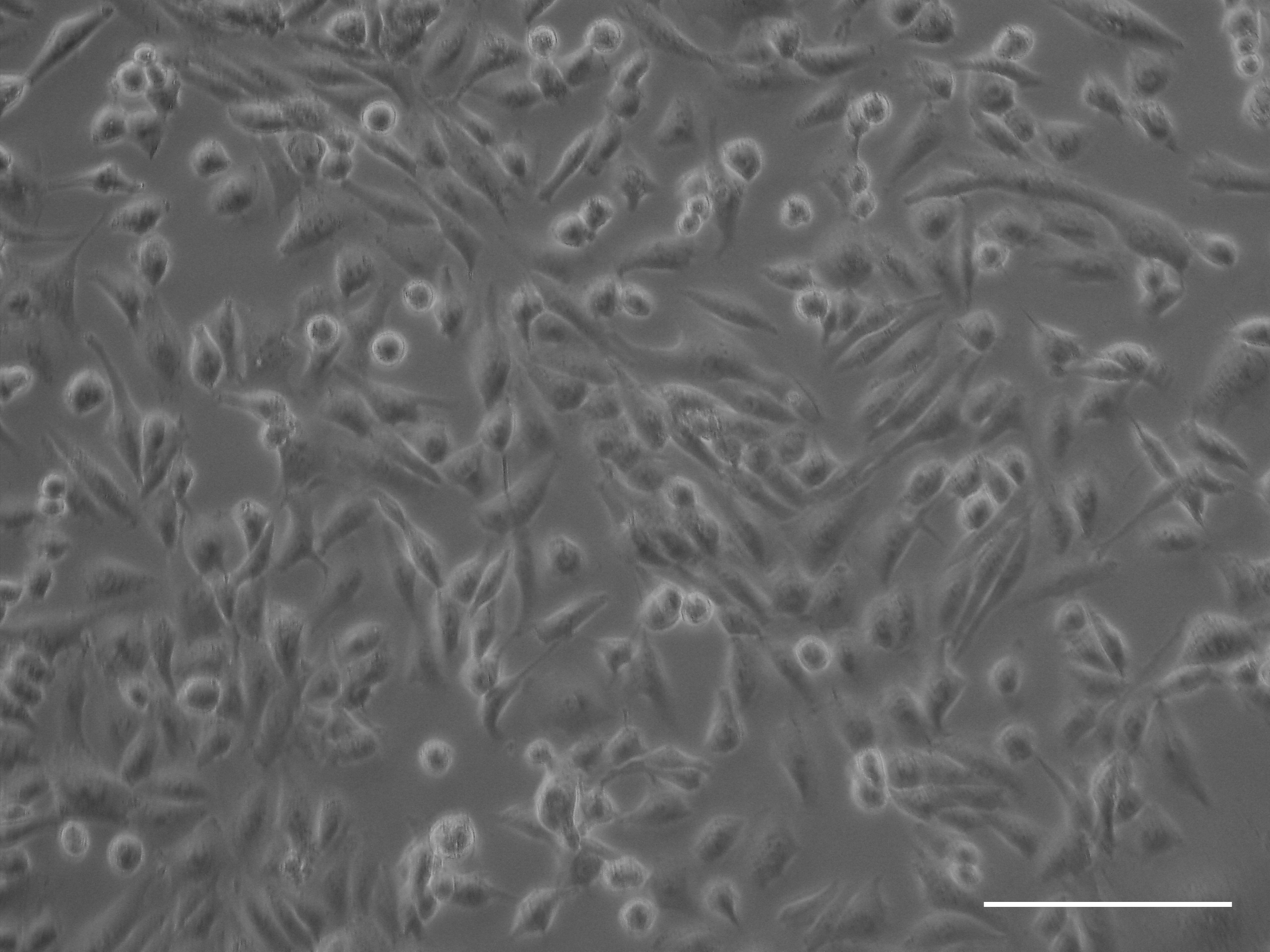

Supplement: S4 File — (ZIP) [file pone.0227851.s004.zip › S4-File_Raw Data and New Figure 3_Cell picture/1st version pictures/TGM2 shRNA.jpg]

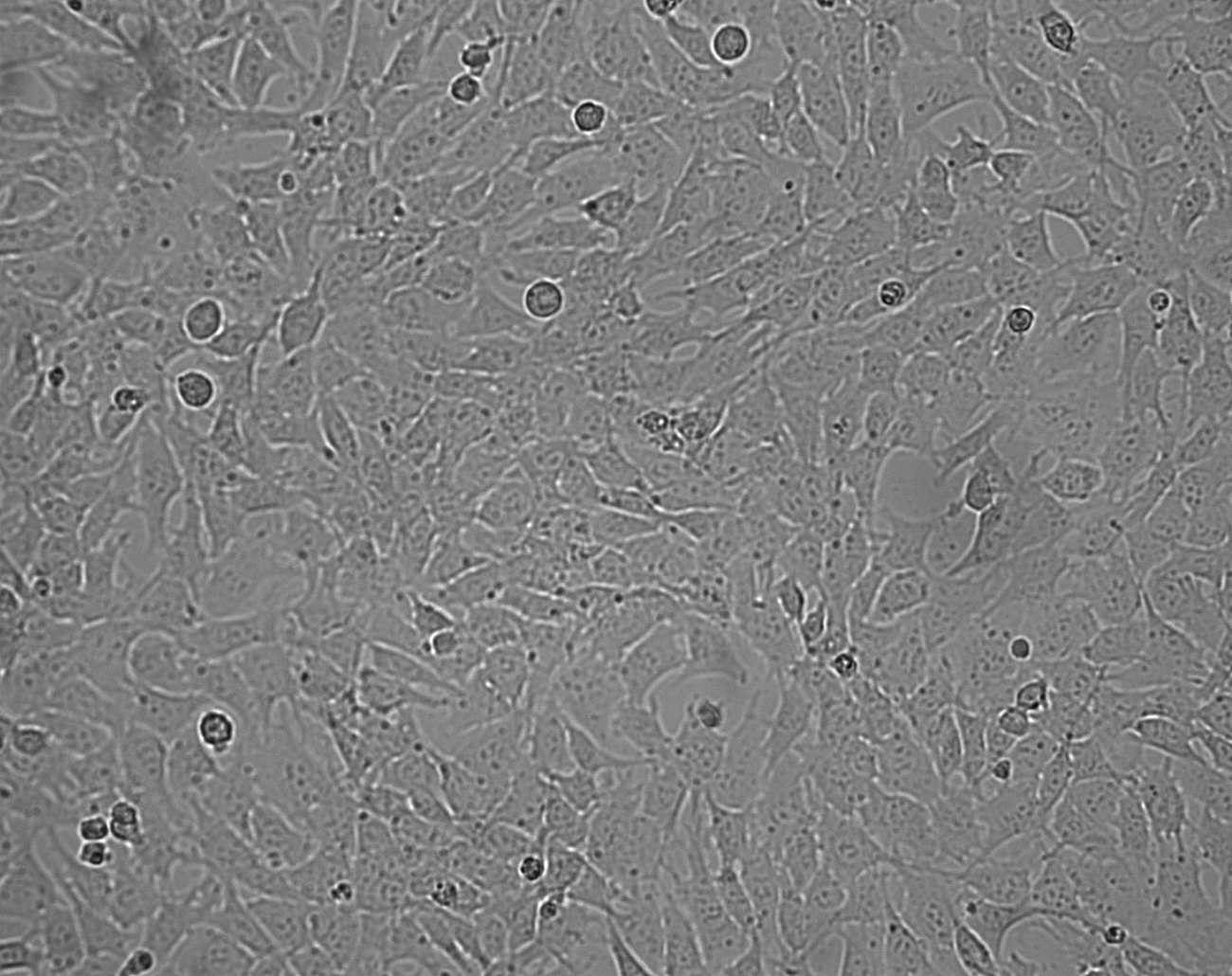

Supplement: S4 File — (ZIP) [file pone.0227851.s004.zip › S4-File_Raw Data and New Figure 3_Cell picture/2nd version pictures/Control.TIF]

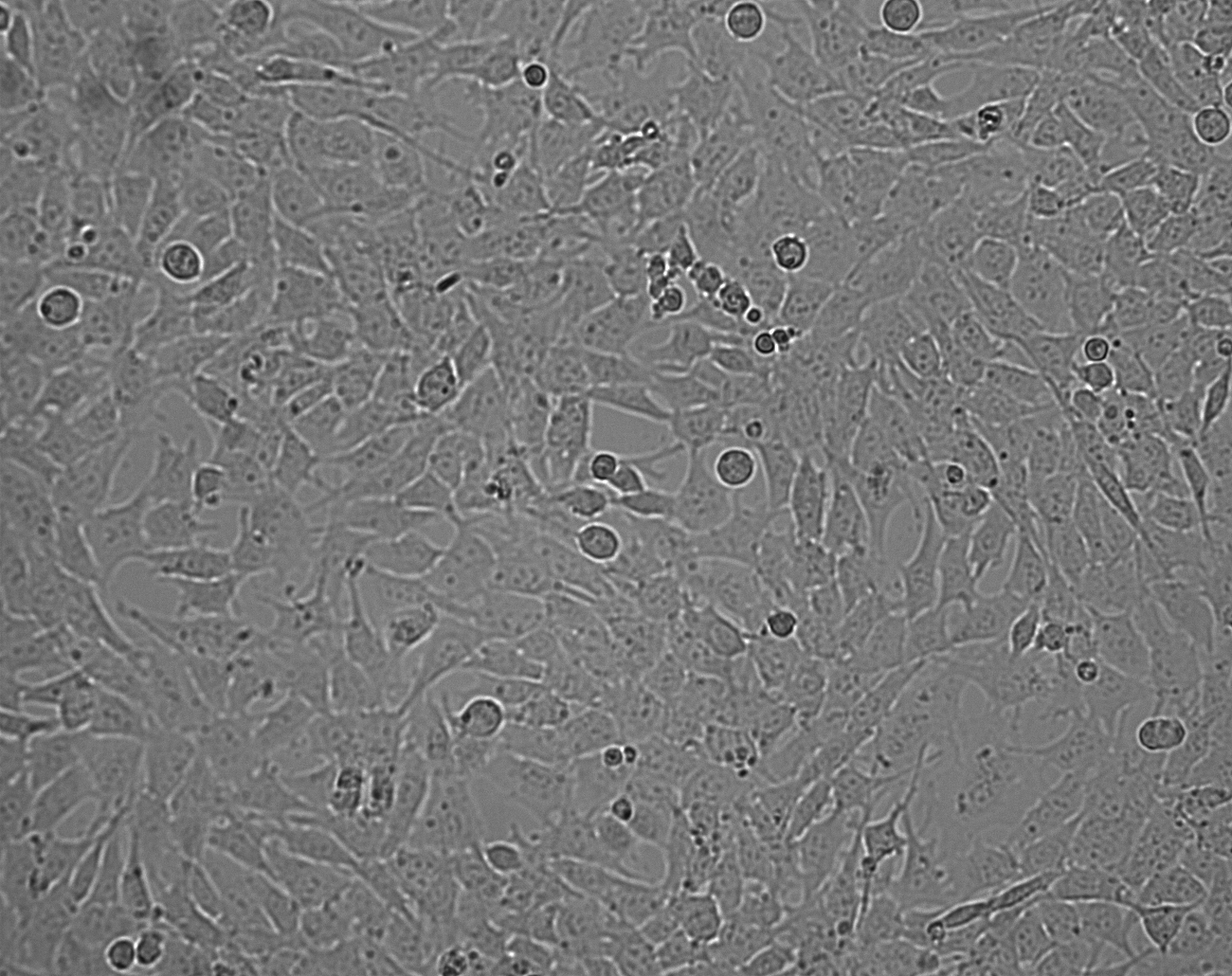

Supplement: S4 File — (ZIP) [file pone.0227851.s004.zip › S4-File_Raw Data and New Figure 3_Cell picture/2nd version pictures/TGM2 shRNA.TIF]

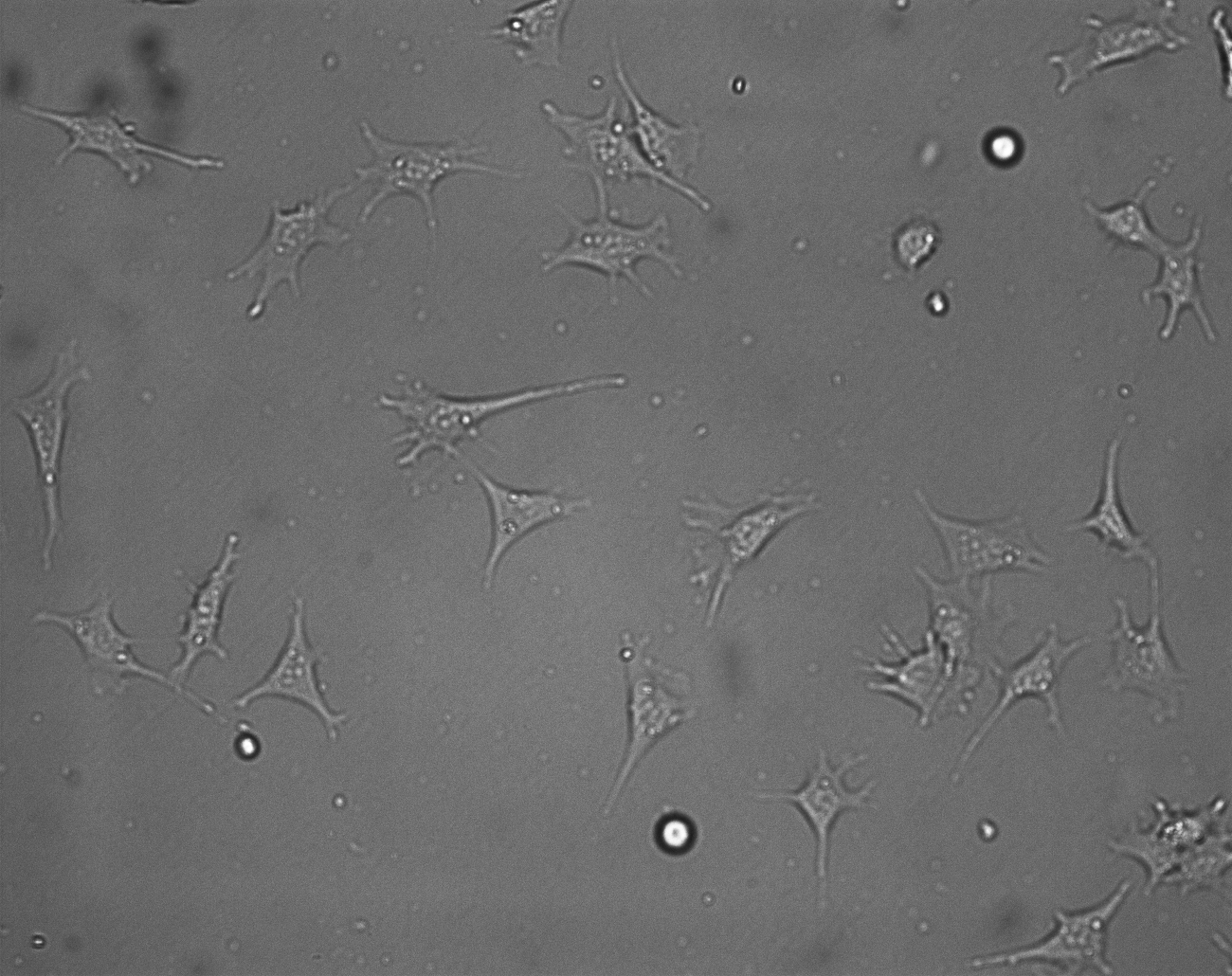

Supplement: S4 File — (ZIP) [file pone.0227851.s004.zip › S4-File_Raw Data and New Figure 3_Cell picture/2nd version pictures/TGM2 shRNA+rapamycin .TIF]

Figure 1G

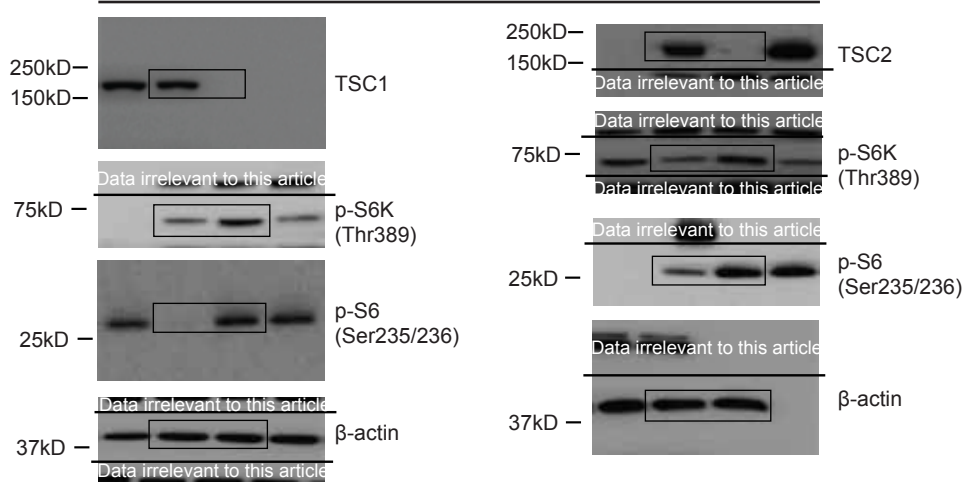

Figure 2B

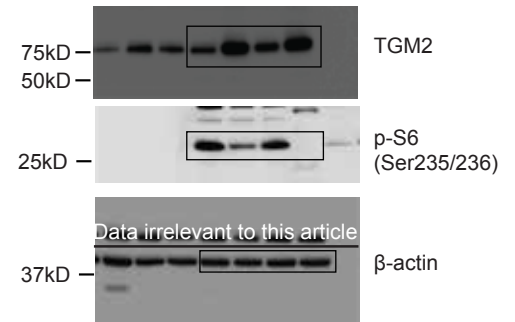

Figure 2D

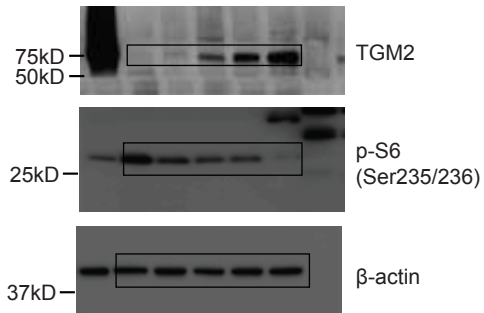

Figure 2F

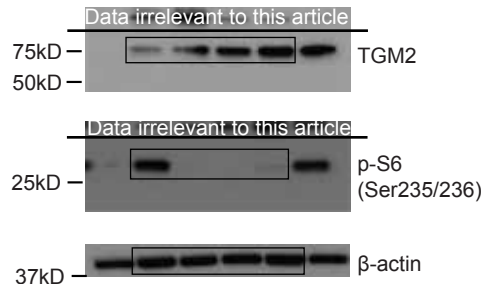

Figure 2G

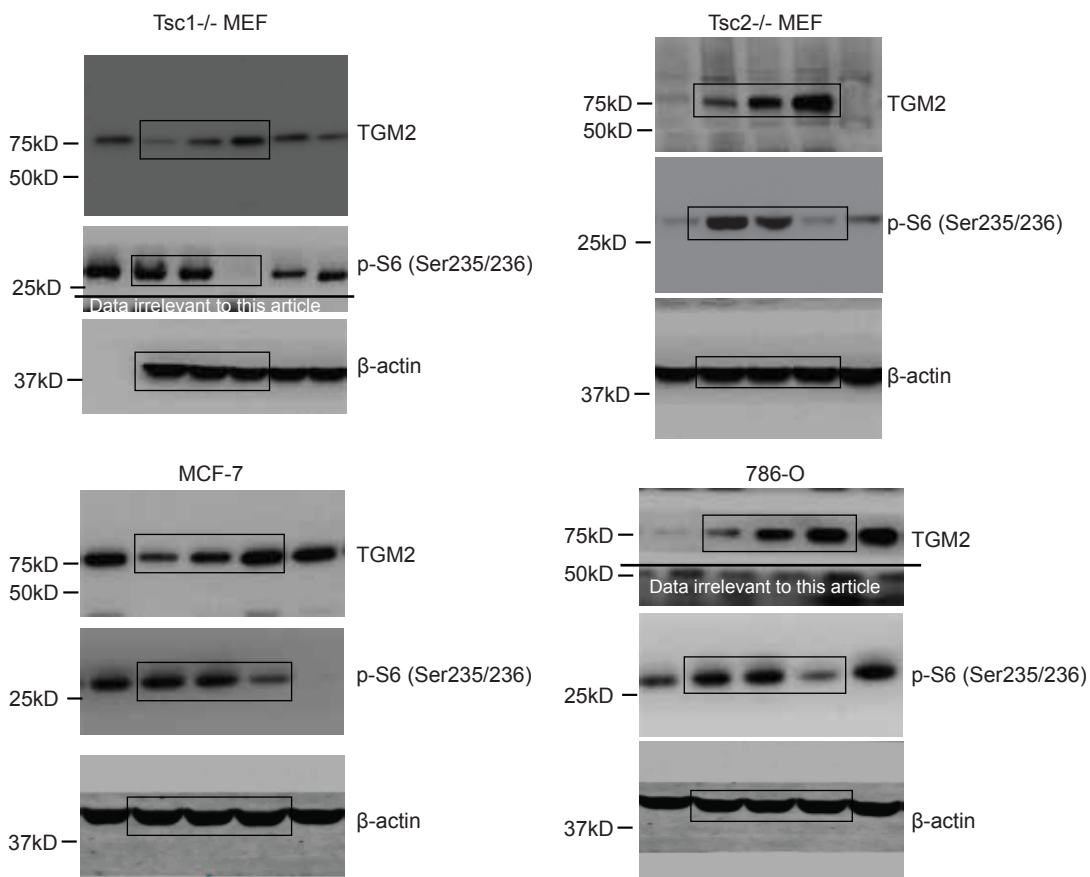

Supplement: S6 File — (PDF) [file pone.0227851.s006.pdf]

Figure 3A

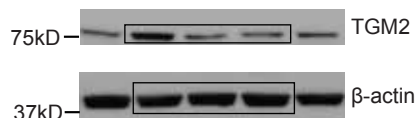

Figure 5A

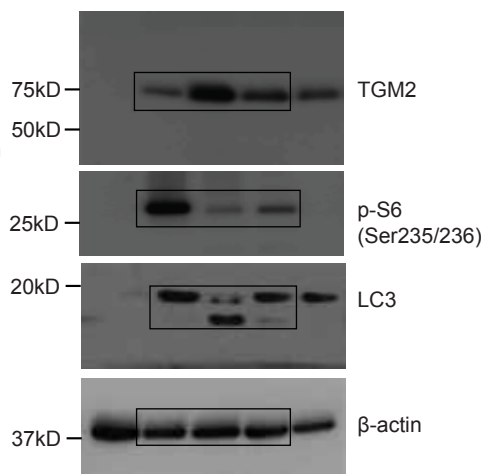

Figure 5B

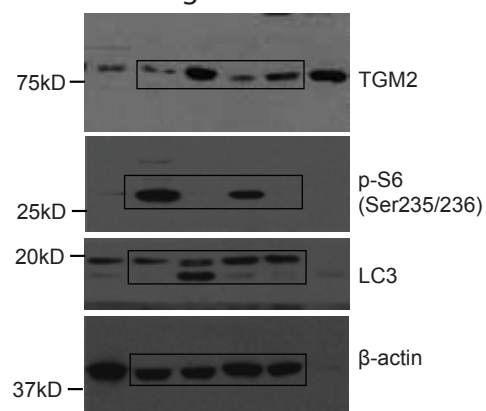

Figure 5C

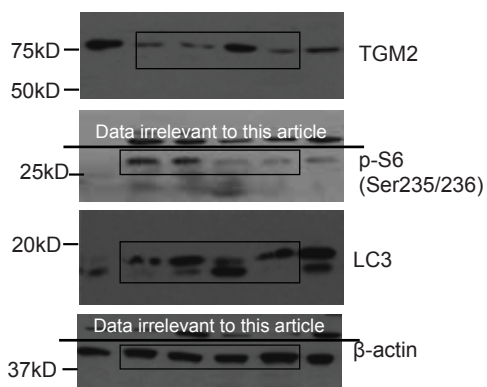

Figure 5D

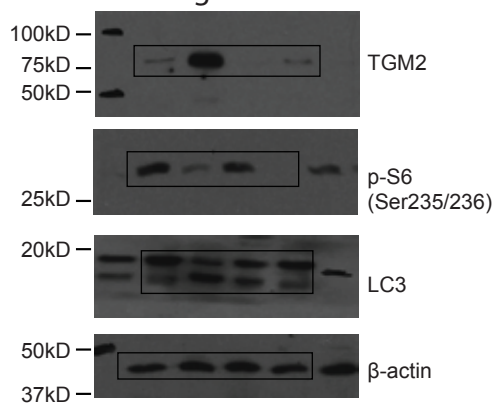

Figure 5E

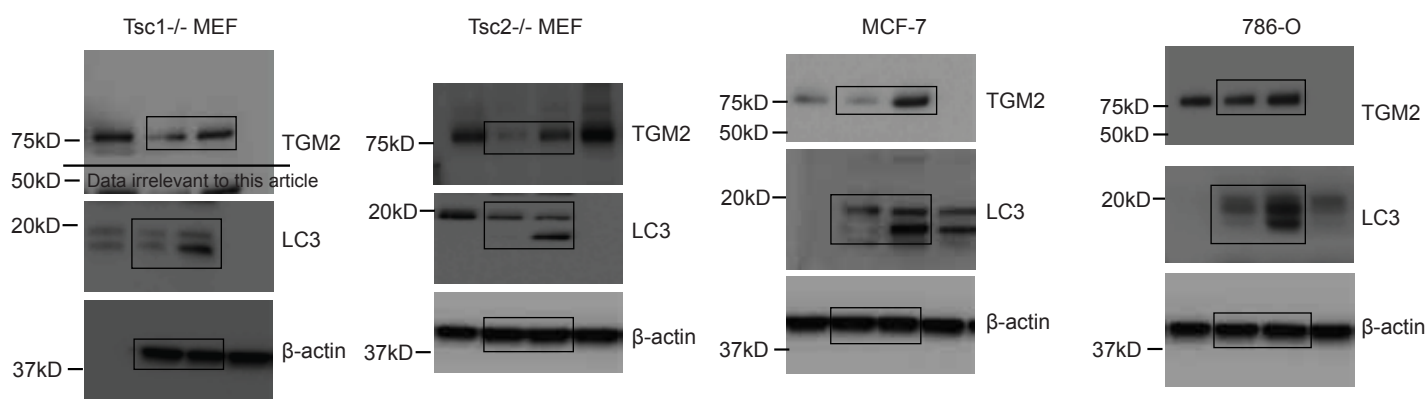

Supplement: S7 File — (PDF) [file pone.0227851.s007.pdf]
